# Heterogeneity of Treatment Effects of Hydrocortisone by Risk of Bronchopulmonary Dysplasia or Death Among Extremely Preterm Infants in the National Institute of Child Health and Human Development Neonatal Research Network Trial: A Secondary Analysis of a Randomized Clinical Trial

**DOI:** 10.1001/jamanetworkopen.2023.15315

**Published:** 2023-05-31

**Authors:** Samuel J. Gentle, Matthew A. Rysavy, Lei Li, Matthew M. Laughon, Ravi M. Patel, Erik A. Jensen, Susan Hintz, Namasivayam Ambalavanan, Waldemar A. Carlo, Kristi Watterberg

**Affiliations:** 1 Department of Pediatrics, University of Alabama at Birmingham; 2 Department of Pediatrics, University of Texas Health Science Center at Houston; 3 Statistics and Epidemiology Division, RTI International, Research Triangle Park, North Carolina; 4 Department of Pediatrics, University of North Carolina at Chapel Hill; 5 Department of Pediatrics, Emory University School of Medicine, Children’s Healthcare of Atlanta, Atlanta, Georgia; 6 Department of Pediatrics, Children’s Hospital of Philadelphia, Philadelphia, Pennsylvania; 7 Department of Pediatrics, Division of Neonatal and Developmental Medicine, Stanford University School of Medicine and Lucile Packard Children’s Hospital, Palo Alto, California; 8 Department of Pediatrics, University of New Mexico Health Sciences Center, Albuquerque

## Abstract

**Importance:**

Extremely preterm infants who develop bronchopulmonary dysplasia (BPD) are at a higher risk for adverse pulmonary and neurodevelopmental outcomes. In the National Institute of Child Health and Human Development Neonatal Research Network (NICHD NRN) Hydrocortisone Trial, hydrocortisone neither reduced rates of BPD or death nor increased rates of neurodevelopmental impairment (NDI) or death.

**Objective:**

To determine whether estimated risk for grades 2 to 3 BPD or death is associated with the effect of hydrocortisone on the composite outcomes of (1) grades 2 to 3 BPD or death and (2) moderate or severe NDI or death.

**Design, Setting, and Participants:**

This secondary post hoc analysis used data from the NICHD NRN Hydrocortisone Trial, which was a double-masked, placebo-controlled, randomized clinical trial conducted in 19 US academic centers. The NICHD HRN Hydrocortisone Trial enrolled infants born at a gestational age of less than 30 weeks who received mechanical ventilation for at least 7 days, including at the time of enrollment, and who were aged 14 to 28 postnatal days. Infants were enrolled between August 22, 2011, and February 4, 2018, with follow-up between 22 and 26 months of corrected age completed on March 29, 2020. Data were analyzed from September 13, 2021, to March 25, 2023.

**Intervention:**

Infants were randomized to 10 days of hydrocortisone or placebo treatment.

**Main Outcomes and Measures:**

Infants’ baseline risk of grades 2 to 3 BPD or death was estimated using the NICHD Neonatal BPD Outcome Estimator. Differences in absolute and relative treatment effects by baseline risk were evaluated using interaction terms in models fitted to the efficacy outcome of grades 2 to 3 BPD or death and the safety outcome of moderate or severe NDI or death by follow-up.

**Results:**

Among the 799 infants included in the analysis (421 boys [52.7%]), the mean (SD) gestational age was 24.9 (1.5) weeks, and the mean (SD) birth weight was 715 (167) g. The mean estimated baseline risk for grades 2 to 3 BPD or death was 54% (range, 18%-84%) in the study population. The interaction between treatment group and baseline risk was not statistically significant on a relative or absolute scale for grades 2 to 3 BPD or death; the size of the effect ranged from a relative risk of 1.13 (95% CI, 0.82-1.55) in quartile 1 to 0.94 (95% CI, 0.81-1.09) in quartile 4. Similarly, the interaction between treatment group and baseline risk was not significant on a relative or absolute scale for moderate or severe NDI or death; the size of the effect ranged from a relative risk of 1.04 (95% CI, 0.80-1.36) in quartile 1 to 0.99 (95% CI, 0.80-1.22) in quartile 4.

**Conclusions and Relevance:**

In this secondary analysis of a randomized clinical trial, the effect of hydrocortisone vs placebo was not appreciably modified by baseline risk for grades 2 to 3 BPD or death.

**Trial Registration:**

ClinicalTrials.gov Identifier: NCT01353313

## Key Points

**Question:**

Does risk of bronchopulmonary dysplasia (BPD) or death modify the effect of hydrocortisone in extremely preterm infants?

**Findings:**

In this secondary analysis of a randomized clinical trial including 799 extremely preterm infants, baseline risk for grades 2 to 3 BPD or death estimated using infants’ gestational age, birth weight, sex, respiratory support, and fraction of inspired oxygen was not associated with either benefit or harm from hydrocortisone exposure.

**Meaning:**

Differences in the association of hydrocortisone with death, BPD, or neurodevelopmental impairment were not identified when analyzed by infants’ baseline risk for BPD or death.

## Teaser

This secondary analysis of a randomized clinical trial investigated whether estimated risk for grades 2 to 3 bronchopulmonary dysplasia or death is associated with the effect of hydrocortisone on the composite efficacy and safety outcomes among extremely premature infants.

## Introduction

Bronchopulmonary dysplasia (BPD) remains the most common serious morbidity of extreme prematurity,^1^ with consequential impact on long-term pulmonary function^2^ and neurodevelopmental outcomes.^3^ There are currently few therapies that are both effective and safe for the prevention or treatment of BPD.^4^ Although dexamethasone therapy reduces the risk for death or BPD,^5^ early exposure increases the risk for adverse neurodevelopment.^6^ Given the safety concerns for dexamethasone therapy, multiple randomized clinical trials^6,7,8,9^ have since investigated whether hydrocortisone therapy is both a safe and effective alternative corticosteroid treatment to reduce risk for BPD.

The recently conducted National Institute of Child Health and Human Development Neonatal Research Network (NICHD NRN) Hydrocortisone Trial compared hydrocortisone with placebo in extremely preterm infants receiving mechanical ventilation between postnatal days 14 and 28.^10^ Hydrocortisone-exposed infants had fewer days with mechanical ventilation up to a postmenstrual age (PMA) of 36 weeks, despite no changes in survival to a PMA of 36 weeks without BPD. However, the impact of treatments to prevent BPD may be affected by baseline risk for BPD. In previously reported metaregressions of randomized clinical trials of corticosteroids for BPD,^11,12^ benefits of corticosteroids outweighed the harms of exposure at specific BPD risk thresholds (eg, >65% risk for BPD or death in the trial population).

The NICHD Neonatal BPD Outcome Estimator uses clinical covariates to estimate infants’ risk at various postnatal time points for the individual outcomes of death; mild, moderate, or severe BPD; or no BPD.^13^ This estimation tool was recently revised^14^ in accordance with the definition for BPD by Jensen et al^3^ and was used in the current analysis, given associations between grades 2 to 3 BPD and adverse long-term outcomes. We hypothesized that in the NICHD NRN Hydrocortisone Trial, infants’ estimated risk for grades 2 to 3 BPD or death was associated with the effect of hydrocortisone on the composite efficacy outcomes of grades 2 to 3 BPD or death and safety outcomes of moderate or severe neurodevelopmental impairment (NDI) or death.

## Methods

We performed a secondary post hoc analysis of the NICHD NRN Hydrocortisone Trial, which was a double-masked, placebo-controlled, randomized clinical trial that enrolled 800 infants from 19 academic centers in the US. Participants were enrolled between August 22, 2011, and February 4, 2018, with follow-up between 22 and 26 months of corrected age completed on March 29, 2020. The centers’ institutional review boards approved the trial, with written informed consent provided by a parent or guardian prior to enrollment. The trial protocol is found in Supplement 1. The primary trial^10^ adhered to the Consolidated Standards of Reporting Trials (CONSORT) reporting guideline. Included infants were born at a gestational age of less than 30 weeks, were receiving mechanical ventilation at study entry between 14 and 28 postnatal days, and underwent mechanical ventilation for at least 7 days. Infants were randomized to either hydrocortisone or placebo treatment over a 10-day course (4 mg/kg per day for 2 days, 2 mg/kg per day for 3 days, 1 mg/kg per day for 3 days, and 0.5 mg/kg per day for 2 days). Both efficacy (BPD or death) and safety (NDI or death) were primary outcomes in the trial.

The definition used for BPD was physiologic moderate or severe BPD assessed at a PMA of 36 weeks,^15^ with the trial preceding the evidence-based BPD grade definition.^3^ Moderate or severe NDI, assessed between 22 and 26 months of corrected age, was defined as any of the following: Bayley Scales of Infant and Toddler Development, third edition, cognitive composite score less than 85 or motor composite score less than 85 (ie, >2 SDs below the scale mean; mean [SD], 100 [15]), cerebral palsy as diagnosed with a Gross Motor Function Classification System level II or greater (on a scale of 0 [normal] to 5 [most impaired]), severe visual impairment, or bilateral hearing impairment with or without amplification.

Data were analyzed from September 13, 2021, to March 25, 2023. The primary outcome for the present analysis used the recently proposed evidence-based BPD grade definition^3^ with the composite outcome including grades 2 to 3 BPD or death. This definition, differing from the use of moderate or severe BPD in the primary trial, was chosen given the higher associated risk for early childhood morbidity at these grades of BPD severity. Additionally, the high and narrow distribution of baseline rate of moderate or severe BPD or death in the primary trial (mean of 89%, with >50% of infants having a risk >92%) precluded an analysis for heterogeneity of treatment effect, as variation in the baseline risk difference across the trial population is necessary to identify subgroups that either benefit or are harmed from exposure.^16^ The publicly available NICHD Neonatal BPD Outcome Estimator,^14,17^ using baseline variables available on postnatal day 14 (earliest postnatal day of randomization), provided estimates of the risk of death and grades of BPD at a PMA of 36 weeks. In this model, the sum of the estimated probabilities of death, no BPD, and grades 1 to 3 BPD total 100%. The sum of an infant’s risk of death and grade 2 or 3 BPD was used to estimate risk of the efficacy outcome. With the NICHD Neonatal BPD Outcome Estimator, we used the following variables to estimate risk: gestational age, birth weight, infant sex, ventilator mode, surgical necrotizing enterocolitis, and fraction of inspired oxygen. While race and ethnicity data were collected, they were not used as covariates in BPD risk estimates, given that these factors are socially constructed and not biological risk factors. Additional comparisons of characteristics and comorbidities of infants in each exposure group were performed by quartiles of baseline risk in the available population. These characteristics included clinical chorioamnionitis, days of mechanical ventilation, medical or surgical treatment for patent ductus arteriosus, early-onset sepsis, late-onset sepsis, and use of open-label dexamethasone.

 To test model performance, in accordance with the Predictive Approaches to Treatment Effect Heterogeneity (PATH) statement recommendations,^18^ BPD or death risk estimates were compared with observed rates of BPD or death via a calibration plot with characterization of distributions of estimated risk by treatment arm. A calibration plot assesses the proficiency of the externally derived risk estimation model to estimate rates of observed outcomes within the trial population at various estimated risks. Additionally, the distribution of risk among enrolled infants was calculated using the extreme quartile risk (mean risk in the highest quartile divided by mean risk in the lowest quartile, and ratio of median to mean risk). Quartiles of baseline risk for BPD or death within the study population were used descriptively to characterize demographic and clinical characteristics by estimated BPD risk.

To describe the magnitude of effect modification,^16^ relative risks (RRs) and risk differences (RDs) for the effect of hydrocortisone vs placebo are shown by quartiles of baseline risk for the outcomes of grade 2-3 BPD or death and moderate or severe NDI or death. For the primary analysis, to determine whether there was an interaction between treatment group and baseline risk for grades 2 to 3 BPD or death as a continuous variable, log-binomial models were used for RR estimation and linear-binomial models for RD estimation with a *P* value estimated for the interaction term.^18^ Relative risk and RD calculations tested variables used for stratification (center and gestational age). Interactions between treatment group and baseline risk of grades 2 to 3 BPD or death were analyzed for both outcomes of grades 2 to 3 BPD or death and moderate or severe NDI or death. We used PROC GENMOD, version 7.15 (SAS Institute Inc), for the estimation of log-binomial and linear-binomial models.^19^ Two-sided *P* < .05 indicated statistical significance.

## Results

A total of 800 infants were enrolled in the NICHD NRN Hydrocortisone Trial (421 boys [52.7%] and 379 girls [47.3%]) (eFigure 1 in Supplement 2), with a mean (SD) gestational age of 24.9 (1.5) weeks and mean (SD) birth weight of 715 (167) g. Of the infants enrolled, 402 were randomized to placebo and 398 to hydrocortisone; all infants were included in the analysis; 1 infant was excluded as mode of respiratory support was not available from postnatal day 14, without which risk for BPD or death could not be estimated. The median day of enrollment was 21 days in both groups (IQRs, 14-28 days for the hydrocortisone group and 15-28 days for the placebo group). The mean estimated probability for grades 2 to 3 BPD or death on postnatal day 14 in the enrolled population was 54% (range, 18%-84%) with a median of 53% (IQR, 45%-65%) (eFigure 2 in Supplement 2). The median extreme quartile risk ratio was 1.82 and the mean extreme quartile risk ratio was 1.88 (ratio of median to mean risk, 0.97). The distribution of risk by quartile was as follows: 18% to 45% for quartile 1, 46% to 53% for quartile 2, 54% to 65% for quartile 3, and 66% to 84% for quartile 4. The calibration plot for the estimated risk for grades 2 to 3 BPD or death using the NICHD Neonatal BPD Outcome Estimator and the observed outcome of grades 2 to 3 BPD or death had a ratio of observed to expected outcomes of 1.09 (eFigure 3 in Supplement 2).

Compared with infants within lower quartiles of baseline risk, infants in the higher quartiles had a lower median birth weight (800 [IQR, 690-925] g in quartile 1 vs 630 [IQR, 530-710] g in quartile 4) (Table 1). More boys were in the placebo-exposed group across all quartiles (eTable 1 in Supplement 2). Regarding clinical characteristics, infants in the higher risk quartiles had higher median fraction of inspired oxygen on postnatal day 14 (35% [IQR, 30%-44%] in quartile 1 vs 60% [IQR, 48%-80%] in quartile 4) (Table 1). Infants within the higher-risk quartiles had more median days of mechanical ventilation exposure (30 [IQR, 21-43] days in quartile 1 vs 46 [IQR, 35-65] days in quartile 4) (eTable 2 in Supplement 2), and open-label dexamethasone exposure occurred more frequently in placebo-exposed infants (156 of 372 [41.9%] vs 150 of 378 [39.7%]) (eTable 3 in Supplement 2).

**Table 1.** Demographic and Clinical Characteristics Used for Risk Estimates by Quartile of Baseline Risk for Grades 2 to 3 BPD or Death

Characteristic	Risk quartile				
	1 (18%-45%) (n = 199)	2 (46%-53%) (n = 200)	3 (54%-65%) (n = 200)	4 (66%-84%) (n = 200)	All (N = 799)
Gestational age, median (IQR), wk	25 (24-26)	25 (24-26)	25 (24-26)	25 (24-26)	25 (24-26)
Birth weight, median (IQR), g	800 (690-925)	720 (618-790)	667 (580-770)	630 (530-710)	690 (600-800)
Sex, No. (%)					
Boys	58 (29.1)	119 (59.5)	121 (60.5)	123 (61.5)	421 (52.7)
Girls	141 (70.9)	81 (40.5)	79 (39.5)	77 (38.5)	378 (47.3)
Highest Fio_2_ on day 14, median (IQR), %	35 (30-44)	40 (35-54)	48 (38-60)	60 (48-80)	45 (35-60)
Respiratory support on postnatal day 14, No. (%)					
Conventional ventilation	4 (2.0)	13 (6.5)	68 (34.0)	165 (82.5)	250 (31.3)
High-frequency ventilation	193 (97.0)	187 (93.5)	132 (66.0)	35 (17.5)	547 (68.5)
Noninvasive positive pressure ventilation	2 (1.0)	0	0	0	2 (0.3)
Surgical NEC, No. (%)	1 (0.5)	7 (0.3)	15 (7.5)	17 (8.5)	40 (5.0)

Abbreviations: BPD, bronchopulmonary dysplasia; Fio_2_, fraction of inspired oxygen; NEC, necrotizing enterocolitis.

**Figure 1.**
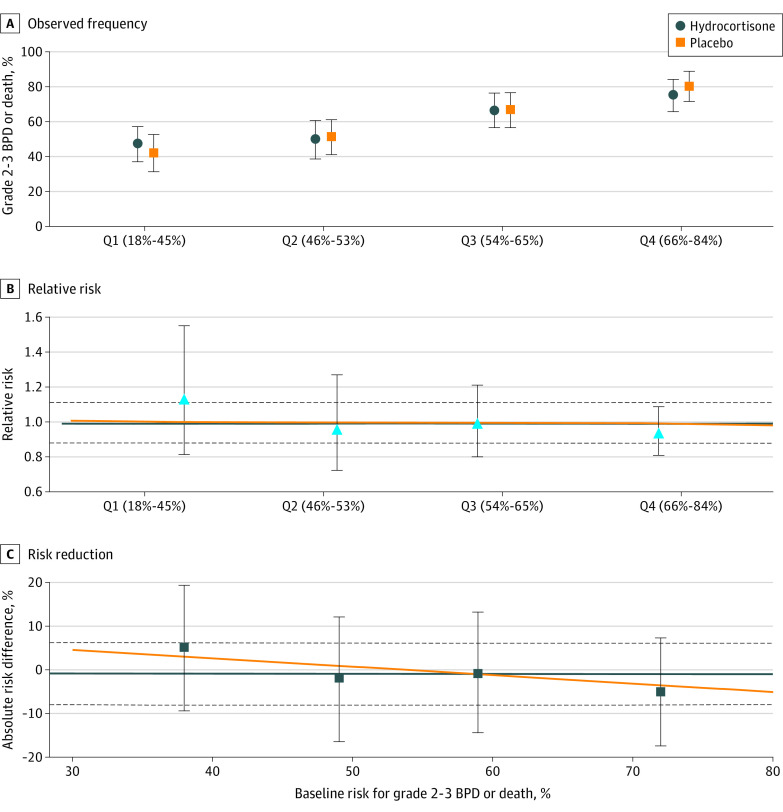
Efficacy Outcome The observed frequency (A), relative risk (B), and risk reduction (C) of grades 2 to 3 bronchopulmonary dysplasia (BPD) or death in the hydrocortisone vs placebo treatment groups are shown by quartile (Q) of baseline estimated risk of grades 2 to 3 BPD or death. Symbols (circles, squares, and triangles) represent estimated values by quartile of baseline risk; error bars indicate 95% CIs. The horizontal orange lines indicate the model estimated results; the horizontal blue lines and dashed lines indicate the overall trial result and 95% CIs, respectively. Grades 2 to 3 BPD or death did not differ by interaction between treatment group and baseline risk for grades 2 to 3 BPD or death.

In the primary analysis for heterogeneity of treatment effect in the efficacy outcome of grades 2 to 3 BPD or death, there was no significant interaction between baseline risk for grades 2 to 3 BPD or death and treatment on a relative or absolute scale (Figure 1). The magnitude of the effect of hydrocortisone ranged from an RR of 1.13 (95% CI, 0.82-1.55) in quartile 1 to 0.94 (95% CI, 0.81-1.09) in quartile 4. The effect of hydrocortisone on the individual components of the composite outcome did not differ by risk quartile (Table 2).

**Table 2.** Outcomes by Quartile of Baseline Risk for Grades 2 to 3 BPD or Death

Outcome	Treatment group, No./total No. (%)		Relative risk (95% CI)	Risk difference (95% CI)
	Hydrocortisone	Placebo		
Grades 2-3 BPD or death				
Quartile 1	51/108 (47.2)	36/86 (41.9)	1.13 (0.82 to 1.55)	0.05 (−0.09 to 0.19)
Quartile 2	44/89 (49.4)	56/109 (51.4)	0.96 (0.73 to 1.27)	−0.02 (−0.16 to 0.12)
Quartile 3	64/97 (66.0)	66/99 (66.7)	0.99 (0.81 to 1.21)	−0.01 (−0.14 to 0.13)
Quartile 4	75/100 (75.0)	80/100 (80.0)	0.94 (0.81 to 1.09)	−0.05 (−0.17 to 0.07)
Overall	234/394 (59.4)	238/394 (60.4)	0.98 (0.88 to 1.10)	−0.01 (−0.08 to 0.06)
Grades 2-3 BPD among survivors				
Quartile 1	50/107 (46.7)	35/85 (41.2)	1.13 (0.82 to 1.57)	0.06 (−0.09 to 0.20)
Quartile 2	43/88 (48.9)	53/106 (50.0)	0.98 (0.73 to 1.30)	−0.01 (−0.15 to 0.13)
Quartile 3	56/89 (62.9)	54/87 (62.1)	1.01 (0.81 to 1.27)	0.01 (−0.13 to 0.15)
Quartile 4	66/91 (72.5)	68/88 (77.3)	0.94 (0.79 to 1.11)	−0.05 (−0.17 to 0.08)
Overall	215/375 (57.3)	210/366 (57.4)	1.00 (0.88 to 1.13)	−0.00 (−0.07 to 0.07)
Death by PMA of 36 wk				
Quartile 1	1/108 (0.9)	1/86 (1.2)	0.80 (0.05 to 12.55)	−0.00 (−0.03 to 0.03)
Quartile 2	1/89 (1.1)	3/109 (2.8)	0.41 (0.04 to 3.86)	−0.02 (−0.05 to 0.02)
Quartile 3	8/97 (8.2)	12/99 (12.1)	0.68 (0.29 to 1.59)	−0.04 (−0.12 to 0.05)
Quartile 4	9/100 (9.0)	12/100 (12.0)	0.75 (0.33 to 1.70)	−0.03 (−0.11 to 0.05)
Overall	19/394 (4.8)	28/394 (7.1)	0.68 (0.39 to 1.19)	−0.02 (−0.06 to 0.01)

Abbreviations: BPD, bronchopulmonary dysplasia; PMA, postmenstrual age.

**Figure 2.**
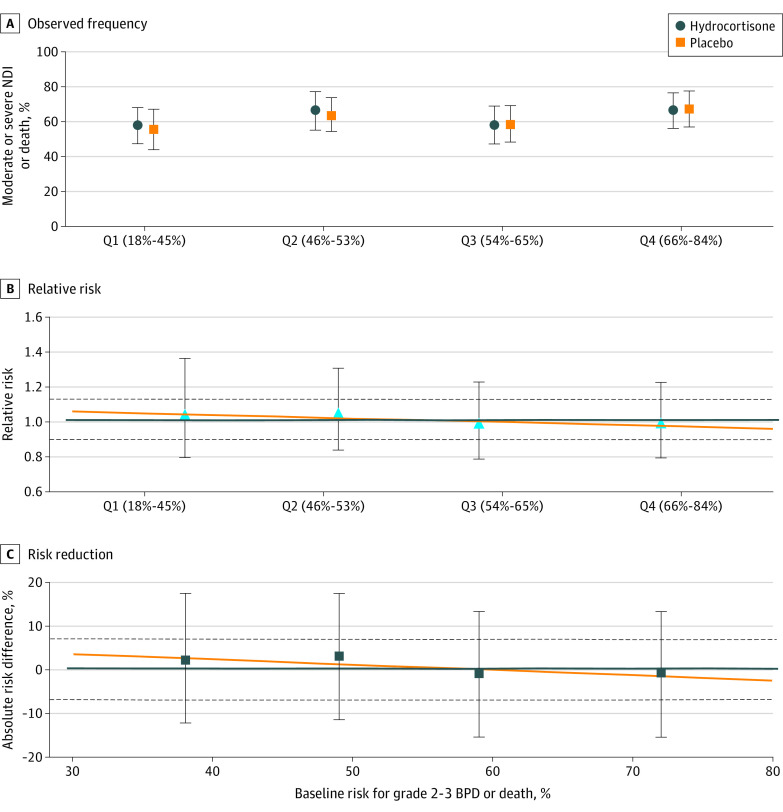
Safety Outcome The observed frequency (A), relative risk (B), and risk reduction (C) of moderate or severe neurodevelopmental impairment (NDI) or death in the hydrocortisone vs placebo treatment groups by quartile (Q) of baseline estimated risk of grades 2 to 3 bronchopulmonary dysplasia (BPD) or death. Symbols (circles, squares, and triangles) represent estimated values by quartile of baseline risk; error bars indicate 95% CIs. The horizontal orange lines indicate the model estimated results; the horizontal blue lines and dashed lines indicate the overall trial result and 95% CIs, respectively. Moderate or severe NDI or death did not differ by interaction between treatment group and baseline risk for grades 2 to 3 BPD or death.

In the analysis of the safety outcome of moderate or severe NDI or death, there was no significant interaction between baseline risk for grades 2 to 3 BPD or death and treatment on a relative or absolute scale (Figure 2). The magnitude of the effect of hydrocortisone ranged from an RR of 1.04 (95% CI, 0.80-1.36) in quartile 1 and 0.99 (95% CI, 0.80-1.22) in quartile 4 (Table 3).

**Table 3.** Outcomes by Quartile of Baseline Risk for Moderate or Severe NDI or Death Between Birth and Follow-up

Outcome	Treatment group, No./total No. (%)		Relative risk (95% CI)	Risk difference (95% CI)
	Hydrocortisone	Placebo		
Moderate or severe NDI or death by follow-up				
Quartile 1	56/97 (57.7)	42/76 (55.3)	1.04 (0.80 to 1.36)	0.02 (−0.12 to 0.17)
Quartile 2	53/80 (66.3)	64/101 (63.4)	1.05 (0.84 to 1.30)	0.03 (−0.11 to 0.17)
Quartile 3	57/90 (63.3)	59/92 (64.1)	0.99 (0.79 to 1.23)	−0.01 (−0.15 to 0.13)
Quartile 4	60/91 (65.9)	60/90 (66.7)	0.99 (0.80 to 1.22)	−0.01 (−0.15 to 0.13)
Overall	226/358 (63.1)	225/359 (62.7)	1.01 (0.90 to 1.13)	0.00 (−0.07 to 0.08)
Moderate or severe NDI				
Quartile 1	51/92 (55.4)	40/74 (54.1)	1.03 (0.78 to 1.36)	0.01 (−0.14 to 0.17)
Quartile 2	48/75 (64.0)	57/94 (60.6)	1.06 (0.83 to 1.34)	0.03 (−0.11 to 0.18)
Quartile 3	45/78 (57.7)	42/75 (56.0)	1.03 (0.78 to 1.36)	0.02 (−0.14 to 0.17)
Quartile 4	39/70 (55.7)	40/70 (57.1)	0.98 (0.73 to 1.30)	−0.01 (−0.18 to 0.15)
Overall	183/315 (58.1)	179/313 (57.2)	1.02 (0.89 to 1.16)	0.01 (−0.07 to 0.09)
Death by follow-up				
Quartile 1	5/95 (5.3)	2/73 (2.7)	1.92 (0.38 to 9.62)	0.03 (−0.03 to 0.08)
Quartile 2	5/80 (6.3)	7/99 (7.1)	0.88 (0.29 to 2.68)	−0.01 (−0.08 to 0.07)
Quartile 3	12/88 (13.6)	17/90 (18.9)	0.72 (0.37 to 1.42)	−0.05 (−0.16 to 0.06)
Quartile 4	21/91 (23.1)	20/90 (22.2)	1.04 (0.61 to 1.78)	0.01 (−0.11 to 0.13)
Overall	43/358 (12.0)	46/359 (12.8)	0.94 (0.64 to 1.38)	−0.01 (−0.06 to 0.04)

Abbreviation: NDI, neurodevelopmental impairment.

## Discussion

In this analysis for heterogeneity of treatment effect in infants enrolled in the NICHD NRN Hydrocortisone Trial, baseline estimated risk for grades 2 to 3 BPD or death was not associated with the effect of hydrocortisone on observed grades 2 to 3 BPD or moderate or severe NDI or death. These findings are consistent with the primary analysis of the NICHD NRN Hydrocortisone Trial, which showed no effect of hydrocortisone on BPD or death or NDI or death.

Our analysis was motivated by the multiple metaregression analyses of clinical trials of postnatal corticosteroids in preterm infants to prevent lung disease.^11,12^ Using summary data from published clinical trials, these analyses have suggested that, in populations with higher rates of BPD, the benefits of postnatal corticosteroids to prevent death or cerebral palsy outweighed the potential harms. However, these metaregression analyses compared data between trials rather than infants within the same trial. By contrast, our analysis used individual-level patient data to assess whether risk of BPD is associated with the efficacy or safety of intervention.

While the overall results of a randomized clinical trial estimate the mean treatment effect for a therapy, an analysis for heterogeneity of treatment effect may identify a specific subpopulation for which there may be greater benefit or harm.^20^ Differences in patient characteristics may inform such heterogeneous treatment effects.^16^ Our analysis was motivated by previously published metaregressions of randomized clinical trials of corticosteroids for BPD^11,12^ that identified specific BPD risk thresholds (eg, >65% risk for BPD or death in the trial population) at which benefits of therapy may outweigh the harms on the outcome of NDI. Additional examples include an analysis of vitamin A therapy in which infants at a lower baseline risk for BPD or death had greater benefit^21^ and the selective benefit for African American infants exposed to inhaled nitric oxide for the reduction of BPD.^22^

Infants enrolled in the NICHD NRN Hydrocortisone Trial were at a particularly high risk for BPD or death. All required mechanical ventilation at 2 weeks’ postnatal age for inclusion in the trial. Therefore, the results of this analysis do not resolve whether there are potential differences in the effect of hydrocortisone in populations exposed at earlier postnatal ages or at different risks for BPD or death. In a randomized clinical trial of low-dose hydrocortisone (cumulative dose, 8.5 mg/kg) initiated within 24 hours after birth,^8^ 49% of placebo-exposed infants died or developed BPD, with a lower rate of death or BPD in hydrocortisone-exposed infants. In a trial of hydrocortisone (cumulative dose of 72.5 mg/kg) with a median enrollment time of postnatal day 10,^7^ 71% of placebo-exposed infants died or developed BPD, with a reduced risk for death at 36 weeks’ PMA in hydrocortisone-exposed infants but not a reduction in the composite of BPD or death. By comparison, the NICHD NRN Hydrocortisone Trial had a much higher risk for BPD or death in the enrolled population, occurring in 83.4% and 86.8% of infants in the hydrocortisone and placebo groups, respectively. These data suggest that hydrocortisone exposure may have a greater effect on BPD or death in trials with a greater proportion of infants at lower risk for BPD or death or in patients exposed at earlier postnatal ages. In addition to these factors, other effect modifiers such as dose, duration of exposure, and differences in inclusion criteria must also be considered when contrasting outcomes between these trials.^5,6,23^

There was no significant interaction for treatment effect by baseline risk for grades 2 to 3 BPD or death and the outcome of moderate or severe NDI or death in this analysis. Whereas previous evidence has supported a higher proportion of adverse childhood outcomes in infants with higher grades of BPD,^3,24^ the rates of moderate or severe NDI or death were similar between quartiles of baseline risk for grades 2 to 3 BPD or death. However, these patient populations may not be comparable as the infants used to derive the evidence-based BPD definition were born at a gestational age of less than 27 weeks without any prespecified respiratory morbidity as required for trial enrollment in the NICHD NRN Hydrocortisone Trial. Moreover, approximately 29% of infants in the cohort that derived the evidence-based definition survived without BPD, whereas approximately 15% of infants in the NICHD NRN Hydrocortisone Trial survived without BPD.

### Strengths and Limitations

Our analysis has several strengths, including being the first analysis, to our knowledge, of heterogeneity of treatment effect in a randomized clinical trial of corticosteroid exposure in extremely preterm infants. Moreover, this analysis assessed heterogeneity of treatment effect for both efficacy (BPD or death) and safety (NDI or death). In clinical practice, baseline risk of BPD or death is commonly used to weigh the benefits and harms of corticosteroid exposure in practice; however, to our knowledge, no individual patient-level data from a clinical trial have been analyzed to study this question. Moreover, as recommended by the PATH statement,^18^ we assessed heterogeneity in terms of overall risk rather than subgroup analyses of 1 variable at a time. Additionally, the BPD risk model was previously validated and accurately predicted the outcome of observed grades 2 or 3 BPD or death, strengthening the conclusions of this analysis.

A significant limitation of this investigation is that the available patient population had both a high and narrow distribution of baseline risk for grades 2 to 3 BPD or death, constraining our ability to analyze treatment effect heterogeneity compared with a population with a wider distribution of baseline risk. Finally, this post hoc analysis of the NRN Hydrocortisone Trial may not have been adequately powered to identify treatment effect heterogeneity; clinically relevant interactions between infant characteristics and treatment effect not identified in this analysis may exist.

## Conclusions

In this secondary analysis for heterogeneity of treatment effect in infants enrolled in the NICHD NRN Hydrocortisone Trial, baseline risk for grades 2 or 3 BPD or death did not modify the treatment effect of hydrocortisone for either observed grades 2 or 3 BPD or death or moderate or severe NDI or death. Similar analyses of other randomized clinical trials of corticosteroid exposure may be warranted to more precisely evaluate whether specific subpopulations of extremely preterm infants are more likely to benefit from or be harmed by systemic hydrocortisone treatment.

## Notes

Supplement 1.
Trial Protocol

Supplement 2. **eTable 1.** Demographic and Clinical Characteristics Used for Risk Estimates by Quartile of Baseline Risk for Grades 2 to 3 BPD or Death and Exposure Group **eTable 2.** Additional Clinical Characteristics by Quartile of Baseline Risk for Grades 2 to 3 BPD or Death **eTable 3.** Additional Clinical Characteristics by Quartile of Baseline Risk for Grades 2 to 3 BPD or Death and Exposure Group **eFigure 1.** CONSORT Flow Diagram **eFigure 2.** Distribution of Predicted Probability of Grades 2 to 3 BPD or Death in the Enrolled Population **eFigure 3.** Calibration Plot of Association of Estimated Probability of Grades 2 to 3 BPD or Death to the Observed Outcome of Grades 2 to 3 BPD or Death

Supplement 3.
Nonauthor Collaborators

Supplement 4.
Data Sharing Statement
