# Error in Table

**DOI:** 10.1001/jamanetworkopen.2023.31067

**Published:** 2023-08-16

In the Original Investigation titled “Heterogeneity of Treatment Effects of Hydrocortisone by Risk of Bronchopulmonary Dysplasia or Death Among Extremely Preterm Infants in the National Institute of Child Health and Human Development Neonatal Research Network Trial: A Secondary Analysis of a Randomized Clinical Trial,”^1^ published May 31, 2023, the subheadings “High-frequency ventilation” and “Conventional ventilation” in Table 1 were inadvertently transposed. This article has been corrected.
