# Supplementary material for: Heterogeneity of Treatment Effects of Hydrocortisone by Risk of Bronchopulmonary Dysplasia or Death Among Extremely Preterm Infants in the National Institute of Child Health and Human Development Neonatal Research Network Trial: A Secondary Analysis of a Randomized Clinical Trial
Source: JAMA Netw Open. 2023 May 31;6(5):e2315315. doi: 10.1001/jamanetworkopen.2023.15315 (PMC10233424; doi:10.1001/jamanetworkopen.2023.15315)
Supplement: Supplement 1. — Trial Protocol [file jamanetwopen-e2315315-s001.pdf]

Protocol

This trial protocol has been provided by the authors to give readers additional information about the work.

This supplement contains the following items:

1. Original protocol, final protocol, summary of changes.

2. The original statistical analysis plan, final statistical analysis plan, and summary of changes are included as part of the protocol, not as a separate document.

A RANDOMIZED CONTROLLED TRIAL OF THE EFFECT OF HYDROCORTISONE ON SURVIVAL WITHOUT BRONCHOPULMONARY DYSPLASIA AND ON NEURODEVELOPMENTAL OUTCOMES AT 18 – 22 MONTHS OF AGE IN INTUBATED INFANTS <30 WEEKS GESTATIONAL AGE

Kristi Watterberg, University of New Mexico
Michele Walsh, Case Western Reserve University
Kathleen Kennedy, University of Texas, Houston
Ron Goldberg, Duke University
Matthew Laughon, University of North Carolina, Chapel Hill
Carl D'Angio, University of Rochester
Abhik Das, RTI
Dennis Wallace, RTI
Conra Lacy, University of New Mexico
Georgia McDavid, University of Texas, Houston
Nancy Morgan, RPh

Version 1.0
Final: May 20, 2011

ABSTRACT:

Bronchopulmonary dysplasia (BPD) remains a leading morbidity of the extremely preterm infant, and prolonged mechanical ventilation is associated with increased risk for BPD. Dexamethasone has been used previously to facilitate extubation and decrease the incidence of BPD; however, due to adverse effects on neurodevelopmental outcomes, the use of this drug has decreased. One cohort study suggests that hydrocortisone (HC) may facilitate extubation. HC has thus far not been associated with adverse neurodevelopmental outcomes in either cohort studies or randomized controlled trials. A recent meta-analysis of postnatal corticosteroid therapy begun after the first week of life suggested that "late therapy may reduce neonatal mortality without significantly increasing the risk of adverse long-term neurodevelopmental outcomes," although the methodological quality of some of the follow-up was acknowledged to be limited.

We propose a randomized controlled trial to study the efficacy and safety of a 10-day tapering course of hydrocortisone treatment for infants <30 weeks estimated gestational age at birth who remain intubated at 14 - 28 days postnatal age. Based on previous Network data these criteria define a population with a risk of death or BPD at 36 weeks postmenstrual age of approximately 65 – 75%. The primary outcome for this study will incorporate both (1) survival without moderate to severe BPD by Network physiologic definition and (2) survival without moderate or severe NDI at 18 – 22 months corrected age. Therefore, the results of this study will be reported only when follow-up data are available unless (1) the trial is stopped early by the DSMC because of strong evidence of benefit or harm, or (2) at the time all subjects have completed treatment the DCC finds a substantial survival benefit favoring hydrocortisone (p<0.001). Individual study assignment will remain masked until the follow-up is completed. Secondary outcomes will include short term measures such as respiratory morbidities and growth at 36 weeks postmenstrual age and long term measures including growth and other outcomes at 18 – 22 months corrected age.

STATEMENT OF THE PROBLEM

Bronchopulmonary dysplasia remains a leading morbidity of the extremely preterm infant, resistant to therapeutic interventions and associated with adverse neurodevelopmental outcomes (1-3). Most studies indicate that its rate of occurrence has not decreased in recent years (4), and several reports suggest that the incidence and/or severity of the disease may be increasing as postnatal glucocorticoid use has decreased (5-7). Although dexamethasone can facilitate extubation and decrease the incidence of BPD (8, 9), such treatment can be associated with increased risk of short- and long-term complications including impaired growth and neurodevelopmental delay (10 – 12). However, because BPD also is associated with increased risk for death and adverse neurodevelopmental outcomes, meta-analyses have suggested that in populations at high risk for death or BPD even dexamethasone therapy may be associated with net benefit (9, 13). Lower doses of dexamethasone may have equal pulmonary benefit with less toxicity (14 – 17); however, because it has a very long biologic half-life, suppresses endogenous cortisol production and disrupts the balance between mineralocorticoid and glucocorticoid in the brain (see below), dexamethasone may still be an inferior glucocorticoid choice for premature infants. Establishment of an alternative therapeutic agent to facilitate extubation may improve respiratory outcomes for these extremely preterm infants at very high risk for BPD, without the adverse effects previously seen with dexamethasone therapy.

HYPOTHESIS

Compared to placebo, hydrocortisone treatment of infants <30 weeks estimated gestational age at birth who remain on mechanical ventilation at 14 – 28 days postnatal age will significantly improve their rate of survival without moderate or severe BPD and will be associated with improvement in survival without moderate or severe neurodevelopmental impairment at 18 – 22 months corrected age.

SPECIFIC AIMS

- To determine whether a 10 day course of hydrocortisone results in a significant increase in survival without moderate or severe BPD;
- To determine whether a 10 day course of hydrocortisone results in a significant increase in the number of infants successfully extubated during treatment;
- To determine whether this treatment affects other short-term outcomes including respiratory morbidities, growth and length of stay;
- To determine whether this treatment affects survival without moderate or severe NDI at 18 – 22 months corrected age; and,
- To determine whether this treatment affects other outcomes, such as growth and pulmonary morbidity, at 18 – 22 months

RATIONALE/JUSTIFICATION/PREVIOUS STUDIES

Glucocorticoid therapy to prevent or treat BPD began with high doses of dexamethasone, a powerful synthetic glucocorticoid with a myriad of predictable adverse effects on short and

long term outcomes, including growth and neurodevelopment (8-12). Lower doses may have equal pulmonary benefit with less toxicity (14-17); however, because it has a very long biologic half-life, suppresses endogenous cortisol production and disrupts the balance between mineralocorticoid and glucocorticoid in the brain (see below), dexamethasone may still be an inferior glucocorticoid choice for premature infants. One cohort study has reported that hydrocortisone may be efficacious in facilitating extubation in preterm infants (18).

Data regarding the effect of neonatal hydrocortisone treatment on neurodevelopmental outcomes are encouraging. A meta-analysis of three RCTs evaluating 333 infants treated for the first two weeks of life with 1-2 mg/kg/day of hydrocortisone showed no difference at 18 – 22 months corrected age in the incidence of cerebral palsy (12% in each group) and a non-significant *increase* in survival without NDI (54% HC vs. 49% placebo) (19). The largest of the included studies (252 infants) showed suggestions of benefit, with a significantly lower incidence of Bayley-II MDI <70 and increased "awareness of object permanence", an early measure of executive function and pre-frontal cortical development (20). At school age (7 – 8 years of age), outcome data from a cohort study comparing 62 infants treated with a 22 day course of hydrocortisone for BPD (median start 19d, initial dose 5mg/kg/day) to 164 non-treated infants who were larger, more mature and less sick showed no evidence of adverse functional or structural (MRI) effects (21). A recent RCT designed to evaluate the effects of hydrocortisone therapy for BPD on brain growth showed that hydrocortisone treatment (a tapering dose over 7 days, starting at 3mg/kg/day, total 17mg/kg) had no adverse effects on brain volumes on MRI at 38 weeks postmenstrual age (n=48, entry from 10 – 21 days postnatal age) (22).

Differences in the actions of dexamethasone and hydrocortisone in the brain may contribute to the observed differences in neurodevelopmental outcomes. Two types of corticosteroid receptors are found in the brain: mineralocorticoid and glucocorticoid. Under basal conditions, cortisol binds preferentially to mineralocorticoid receptors in the brain; at times of higher stress, cortisol also binds to glucocorticoid receptors (23, 24). Dexamethasone is a powerful synthetic glucocorticoid with no mineralocorticoid activity and a very long biologic half-life. It suppresses endogenous cortisol production, leaving mineralocorticoid receptors unoccupied, thereby creating a "chemical adrenalectomy" (23, 24). A lack of cortisol binding to mineralocorticoid receptors has been shown in animal studies to result in neuronal apoptosis. This apoptosis occurs whether the lack of cortisol binding is the result of a cortisol deficiency produced by adrenalectomy or the result of dexamethasone administration (25, 26). Further, concurrent administration of corticosterone (the cortisol equivalent in rats) with dexamethasone has been shown to protect against dexamethasone-induced apoptosis (25, 26).

Corticosteroid receptors are found in high density in the hippocampus, an area of the brain critical for learning and memory (26-29). Individuals born preterm have reductions in hippocampal volume compared to term (27-29), and hippocampal gray matter reduction has been associated with memory deficits in adolescents born prematurely (28). In small cohort studies, preterm infants treated with dexamethasone have been found to have decreased grey matter and/or hippocampal volume compared to untreated controls (30-32); however, those treated with hydrocortisone have shown no reduction in volume or increase in lesions vs. untreated controls (21, 33).

Thus, there is both biologically plausible basis for preferring hydrocortisone over dexamethasone and clinical evidence in preterm infants that hydrocortisone does not impair long term neurodevelopmental outcomes whereas dexamethasone can do so. In addition, there is

preliminary evidence from a small cohort study that hydrocortisone is equally efficacious as dexamethasone in facilitating extubation in preterm infants. Therefore, we propose a randomized, masked, placebo-controlled trial to study (1) the efficacy of hydrocortisone in facilitating extubation and thereby increasing survival without moderate or severe BPD and (2) the safety of HC for this use, by assessing the effect of hydrocortisone on survival without moderate or severe NDI at 18 – 22 months adjusted age.

METHODS/PROCEDURES

(1) Study design: Randomized, masked placebo-controlled trial.

(2) Study population: Patients eligible for this study will be infants between 14 – 28 postnatal days who:

(a) are <30 weeks estimated gestational age, to be randomized in two strata: ≤ 26^6 and 27^0 – 29^6 weeks);

(b) were inborn at an NRN site or were admitted to an NRN site ≤ 72 hours postnatal age;

(c) have received ≥ 7days of mechanical ventilation;

(d) are receiving mechanical ventilation through an endotracheal tube .

This population is at very high risk for death or moderate to severe BPD, based on previous NRN data (approximately 65 – 75%, see data below), and is a population for whom the most recent Cochrane review suggests it is reasonable to consider corticosteroid therapy (9). Deferring study entry until 14 days postnatal age will avoid the period of highest risk for spontaneous intestinal perforation (34).

Data provided by Dr. Das for the two year period 2006 – 2007, using the physiologic definition of moderate to severe BPD, i.e., supplemental oxygen and/or positive pressure ventilation at 36 weeks postmenstrual age showed that:

Of infants who received ≥ 7 days of mechanical ventilation in the first 14 days, the percentage surviving without BPD was:

```
 401-999g BW: 368/1482(24.83%)
1000-1500 BW: 166/399 (41.80%)
Total (401-1500 BW): 534/1881(28.4%)
```

Of infants who received ≥ 7 days of ventilation in the first 28 days, the percentage surviving without BPD was:

```
 401-999g BW: 457/1528(29.91%)
1000-1500 BW: 229/490 (46.73%)
Total (401-1500 BW): 686/2018 (33.99%)
```

Of infants who received ≥ 14 days of ventilation in the first 28 days, the percentage surviving without BPD was:

```
  401-999g BW: 292/1249(23.38%)
 1000-1500 BW: 57/222 (25.68%)
 Total (401-1500 BW): 349/1471(23.73%)
```

In addition, unpublished data from the SUPPORT trial showed that of the 526 infants who were on mechanical ventilation at 14 days, 372 (71%) died or developed BPD.

We will include infants who have failed extubation because of apnea, because they too are at risk for BPD from prolonged intubation. HC may promote successful extubation in these infants by decreasing ongoing lung inflammation and capillary leak and improving FRC.

(3) Exclusions:

(a) Major congenital anomalies

(b) Decision to limit support

(c) Indomethacin or ibuprofen treatment within 48 hours of study drug

(d) Previous corticosteroid treatment for BPD

(e) Hydrocortisone treatment for hypotension in the first week of life is common (35) and will not be an exclusion; however, infants will be excluded if they have received hydrocortisone:

(i) for ≥ 14 cumulative days OR

(ii) within 7 days of study entry.

(f) Enrolled in a conflicting clinical trial

(4) Study procedures

(a) Ascertainment of study eligibility: Prior to the 14th postnatal day, the clinical care team will be asked whether they plan to extubate a potentially eligible infant within the next 24 – 48 hours. If so, approach for parental consent will be deferred for that time period. If the infant is not extubated by the 14th postnatal day, or if he/she fails extubation within the study window, AND if the attending physician agrees to the patient's entry into the study, then the parents will be approached for consent.

(b) Randomization procedures:

i. Infants will be randomized 1:1 to the two arms using a stratified, permuted block design with strata for gestational age as described above within center.

ii. After parental consent is obtained, the NRN coordinator at each center will randomize subjects by telephoning the Data Coordinating Center at RTI International, Research Triangle Park, NC; the randomization system will be available 24 hours per day, 7 days per week.

iii. Pharmacy personnel will prepare and dispense study drug in accordance with procedures described in the study manual. HC and normal saline placebo are indistinguishable in appearance.

(c) Therapeutic intervention: The infant will be randomized to saline placebo or hydrocortisone sodium succinate for intravenous administration (unpreserved, Solu-Cortef plain, Pfizer®, reconstituted with unpreserved normal saline to avoid exposure to the benzyl alcohol contained in preserved diluents), to be administered either intravenously or orally if no intravenous line is available (route of delivery will be recorded on drug administration forms), at the same dose, and tapered as follows:

4mg/kg/day ÷ q 6 hours x 2 days, then
2mg/kg/day ÷ q 6 hours x 3 days; then
1mg/kg/day ÷ q 12 hours x 3 days; then
0.5mg/kg/d as a single dose x 2 days

Rationale for dosing: A dose of 4mg/kg/day is intended to reach anti-inflammatory plasma concentrations (36), and is similar to the starting hydrocortisone dose in the previously reported cohort study (5mg/kg/day) (18) and the dose of dexamethasone given in the DART study (starting at 0.15mg/kg, ≅ 4 – 6mg/kg/day hydrocortisone) (15). The length of therapy is shorter than the previous hydrocortisone cohort study (18), but equal to the DART study, consistent with an overall goal of giving lower doses of glucocorticoid for shorter periods of time (9).

Need to taper study drug if parents withdraw consent during therapy: Sudden withdrawal of glucocorticoid therapy may precipitate acute adrenal insufficiency; therefore, language will be included in the consent form as follows: ***"If you should decide to withdraw your child from the study during the study drug administration period, for reasons of safety, your child will have the study drug gradually decreased until off".*** If parents withdraw consent for their infant to participate in this trial during the period of study drug administration, the study will NOT be unblinded, and the following taper protocol will be utilized:

- If up to 8 doses have been given (4mg/kg/day dosing level): drop to the next dose level x 2 days, then subsequent dose x 3 days, then lowest dose for 2 days.

- If 9 - 12 doses have been given (first day of the 2mg/kg/day dose): stay at this dose for one more day (total of 8 doses at this dosing level), then drop to the subsequent dosing level (1mg/kg/day) for 3 days, then lowest dose for 2 days.

- If 13 - 16 doses have been given (day 2 of the 2mg/kg/day dose): stay at this dose through dose #16, then drop to the subsequent dosing level (1mg/kg/day) for 3 days, then lowest dose for 2 days.

- If 17 - 20 doses have been given (day 3 of the 2mg/kg/day dose): drop to next dosing level (1mg/kg/day) for 3 days, then lowest dose for 2 days.

- From dose 21 on, taper as described in the study protocol.

If parents refuse to have the study drug weaned by this accelerated taper even after discussion with investigator or clinical attending physician, the blind may be broken to determine the best course of action.

<u>(d) Extubation:</u> Infants at this age may not have blood gases obtained regularly. Therefore, extubation criteria include information available without a blood gas, and were targeted toward the lower end of the criteria in the SUPPORT trial. Extubation must be attempted within 72 hours of starting study drug and within 24 hours after meeting the following criteria: the FiO2 is <0.40 to maintain a saturation of 88%, the mean airway pressure is < 8, and the infant is hemodynamically stable (as defined for the SUPPORT trial: "clinically acceptable blood pressure and perfusion in the opinion of the clinical team"). The infant may be extubated from higher settings at the discretion of the attending physician. If the infant does not meet these criteria within 72 hours, he/she will be reassessed every 24 hours during the intervention period and extubation will be attempted within 24 hours of those criteria being met. If the infant **is intentionally extubated per study criteria** and subsequently re-intubated, further attempts at extubation will be at the discretion of the clinical care team. [**<u>Note</u>**: inadvertent extubation and subsequent re-intubation are not included in this statement. Such an infant will still be eligible for extubation per study protocol.]

<u>(e) Concurrent therapy with indomethacin/ibuprofen:</u> As stated above, infants who have received indomethacin or ibuprofen within 48 hours are not eligible for the study at that time. Spontaneous gastrointestinal perforation primarily occurs during the first 14 days of postnatal life; therefore, these infants should be at low risk for this event (34). However, to minimize any remaining risk, if a patient in this study is determined by the clinical care team to have developed a PDA during the intervention period which <u>must</u> be treated before study drug is completed, the attending physician <u>may</u> choose to stop the study drug prior to treatment with indomethacin or ibuprofen. If upon discontinuing study drug the patient becomes hypotensive and oliguric with an electrolyte profile consistent with adrenal insufficiency, the attending physician <u>may</u> choose to institute open-label hydrocortisone treatment or other clinically-indicated therapy for the hypotension.

(f) Clinical deterioration during study drug weaning: In this high-risk population, it is anticipated that some infants will experience clinical deterioration during or immediately following study drug therapy for unrelated causes, such as nosocomial sepsis, NEC or pneumonia. Although we do not anticipate that infants will experience clinical deterioration due to adrenal suppression during this short course of hydrocortisone, we will monitor for development of clinical signs of adrenal insufficiency during and immediately after treatment with study drug (see adverse event monitoring below). If an attending physician believes that the infant is experiencing hypotension with inadequate perfusion and oliguria (i.e., shock) due to adrenal insufficiency, a clinically-indicated blood specimen may be drawn for cortisol and the attending physician may institute therapies that he/she feels are clinically necessary for the care of the infant. Study drug should not be stopped for this occurrence. These events will be recorded on a study form created specifically for this study (to include above factors and attending physician judgment regarding the etiology of the event), and the event rate for such clinical deterioration in each group will be monitored by RTI and forwarded to the DSMC with other regularly transmitted study data.

(5) Post-extubation respiratory care

(a) Respiratory support: Each center currently cares for these infants with techniques they feel appropriate, whether CPAP, CPAP with IMV, high-flow nasal cannula or other. This study will not prescribe the technique to be used. Because this is an RCT and because enrollment will be stratified by center and study treatment will be masked, the center-specific and attending-specific techniques should be balanced in both treatment groups equally over the course of the trial and therefore not introduce bias.

(b) Open-label glucocorticoid therapy

(i) Infants who remain successfully extubated are not to be treated with open-label glucocorticoids as therapy for BPD. This will be a protocol violation.

(ii) Infants who are not extubated during the study treatment period or who are subsequently re-intubated may be treated with open-label dexamethasone after ≥ 4 days following the last dose of study drug. Open-label dexamethasone will be encouraged to be prescribed as described by Doyle (15). Further treatment with HC for prevention/treatment of BPD will be considered a protocol violation. The rationale for this plan is as follows: (1) HC has not been demonstrated in an RCT to be efficacious for extubation, whereas dexamethasone has (8, 9, 15); (2) this design will permit a true RCT of HC without open-label contamination of the placebo group. At the same time, we will not be denying treatment to the group of infants that meta-analysis suggests derive net benefit from treatment with dexamethasone (13)

(6) Definition of successful extubation: An infant who remains extubated for at least one week, including at least 3 days (72 hours) after the last dose of study medication, will be considered to be successfully extubated in response to this treatment.

(7) Laboratory specimens: none

(8) Analysis of study outcomes

(a) Study specifications and definitions

(i). Analysis will be by intention to treat.

(ii) Moderate or severe BPD is defined as the need for supplemental oxygen by NRN physiologic definition at 36 weeks estimated gestational age. This includes an oxygen withdrawal test for infants receiving <0.30FiO2, as described in the study manual of procedures. For this study, this determination will be made between 36° to 36^{6} weeks.

(iii) Infants discharged on oxygen prior to 36 weeks gestation will be evaluated at 36 weeks gestational age at a follow-up clinic visit, a home visit or by telephone if necessary.

(iv) Outcomes assessed at 18 – 22 months corrected age will be the standard NRN follow up assessments: demographics, medical history including neurosensory outcomes, physical and neurologic examination, neurodevelopmental and behavioral assessment with the Bayley Scales of Infant Development-III, and social/behavioral development with the Brief Infant Toddler Social Emotional Assessment (BITSEA).

(v) “Severe neurodevelopmental impairment” will be defined as (1) a BSID-III cognitive score more than 2 SD below the mean (<70); or (2) a Gross Motor Function classification score of 3 – 5 (indicating moderate to severe cerebral palsy); or (3) hearing impairment requiring hearing aids; or (4) blindness.

(vi) “Moderate neurodevelopmental impairment” will be defined as (1) BSID-III cognitive score 1 – 2 SD below the mean (70 – 84); OR (2) a GMFCS of 2 (indicating mild cerebral palsy); OR (3) a hearing impairment without amplification.

(vii) Because the primary outcome for this study includes evaluation at 18 – 22 months, earlier study outcomes will not be reported unless the DSMC stops the trial prematurely because of strong evidence of benefit or harm, or at the time all subjects have completed treatment the DCC determines a substantial mortality benefit favoring hydrocortisone that is significant at $p<0.001$.

(b) Primary outcome measure and analytic approach: The primary outcome for this study will include both a measure of efficacy (improvement in survival without physiologically defined moderate to severe BPD) and safety (survival without moderate or severe NDI at 18 – 22 months corrected age). Because the primary study outcome will not be known until evaluation of the outcomes at 18 – 22 months corrected age, BPD outcome data will not be released until after this time.

We propose that if hydrocortisone treatment is efficacious in improving survival without moderate or severe BPD, this treatment will also result in an improvement in survival without NDI, since BPD is a risk factor for both mortality and adverse neurodevelopmental outcomes. Meta-analysis of early, low-dose hydrocortisone trials showed a 5% overall increase in survival without major NDI (19). The largest of those trials showed a significant improvement in the percentage of infants with MDI <70 (27% vs. 37%) and a 4 percentage point improvement in survival without NDI (0.68 (0.41–1.10)), even in the absence of a significant effect on BPD (20). No previous RCT or cohort study of hydrocortisone using 5mg/kg/day or less has shown adverse neurodevelopmental effects associated with its use (18-22). Meta-regression has shown that even dexamethasone, with its numerous short and long term adverse effects, provided net benefit for the outcome of death or cerebral palsy for infants with an *a priori* risk of BPD or death of >65% (13).

This study will provide a sequential evaluation of the composite hypothesis that (i) administration of hydrocortisone has efficacy benefit in that it reduces the risk of death or BPD at 36 weeks; and (ii) administration of hydrocortisone has an acceptable long-term safety profile with respect to death or NDI at 18 to 22 months. Specifically, the primary outcome for this study will include both a measure of efficacy (improvement in survival without BPD) and safety (survival without major NDI at 18 – 22 months corrected age). This composite hypothesis will be evaluated sequentially. First, point and interval estimates of the adjusted relative risk obtained from a robust Poisson regression model controlling for center and gestational age will be used to test the hypothesis that infants treated with hydrocortisone had a lower risk of death or BPD at 36 weeks than infants treated with placebo. If this test of efficacy indicates benefit of the hydrocortisone arm then the safety of the treatment will be evaluated descriptively through an assessment of the risk to benefit ratio of the hydrocortisone treatment. Specifically, this safety outcome will be considered a “success” if either (1) the point estimate of risk of death/NDI is lower on the hydrocortisone arm than on the control arm, or (2) there is an increase in risk on the hydrocortisone arm, but the lower limit of a one-sided 95% confidence interval for the ratio of increased benefit for BPD to increased risk for NDI is greater than 4. In other words, for every additional 4 infants surviving without BPD, no more than 1 additional infant would experience death/NDI.

(c) Power calculations and sample size:

Consistent with the approach for evaluating the composite hypothesis defined above, the sample size calculations were conducted in two stages. First, standard power analysis software was used to assess the sample size required to demonstrate a difference in death or BPD at 36 weeks on the two treatment arms. Second, simulation analyses were used to assess the probability of success for the safety outcome based on either of

the two criteria defined above. The empirical probability of success for the descriptive analyses used to evaluate the safety outcome is analogous to the power of a formal hypothesis test.

As summarized in the subsections below, a sample size of 800 subjects will be adequate to provide the study with a power of 80% for the efficacy hypothesis and a probability of greater than 80% of a successful safety evaluation. With the population available (below), this sample size should allow completion of enrollment in less than 3 years, will give a clear answer regarding the efficacy of hydrocortisone for improving survival without moderate or severe BPD, and will allow a reasonable assessment of safety and potential benefit at 18 – 22 months corrected age.

(i) Efficacy:
Empirical studies of the effect of alternative analytic approaches for binary outcomes (e.g. tests of differences in proportions, logistic regression, Mantel-Haenszel tests, and Poisson regression models) indicate that unless the study has both substantial imbalance of sample sizes across strata and the substantial stratum-specific heterogeneity of treatment effect, power calculations for the various approaches can reasonably be approximated by standard tests for differences in proportions. Consequently, we used standard software to estimate sample sizes for a power of 0.80 under the assumption that the risk of death or BPD at 36 weeks on the control arm was in the range of 65% to 75% (or alternatively that the probability of survival without BPD was in the range of 25% to 35%). Considering a 10 percentage point difference in survival without BPD to be clinically significant, improving survival without BPD from approximately 25% in this population (historical NRN data) to 35% with a power of 0.80 would require a sample size of 658 infants. If the incidence of survival without BPD in the placebo group has improved over time to 35%, we can achieve a power of 0.80 to detect an increase to 45% with a sample size of 752. A sample size of 800 subjects will be adequate to assure that the study has a power of at least 80% to detect a 10 percentage point improvement in outcome regardless of the baseline incidence of survival without BPD, with a margin for a small loss in power if the treatment effect has modest heterogeneity across strata.

(ii) Safety: In view of the previous data suggesting possible benefit from hydrocortisone treatment, we anticipate that hydrocortisone treatment will be associated with improved survival without moderate or severe neurodevelopmental impairment (defined “NDI”) in this population. However, we do not anticipate demonstrating a statistically significant benefit with this sample size. Instead, we postulate based on previous data that hydrocortisone will provide a 3% improvement in survival without NDI (19,20), and will consider this outcome successful if either (1) the risk of death/NDI is lower on the hydrocortisone arm than on the control arm, or (2) there is an increase in death/NDI in the hydrocortisone arm, but the lower limit of a one-sided 95% confidence interval for the ratio of increased benefit for BPD to increased risk for NDI is greater than 4. In other words, for every additional 4 infants surviving

without moderate or severe BPD, we would have 95% confidence that no more than 1 additional infant would experience death/NDI. For example, if we achieve the successful outcome of an increase of 10% in survival without moderate or severe BPD, we would hypothesize also achieving an increase of 3% in survival without NDI, but would only define the treatment as a success if there were no more than a 2.5% increase in death or NDI in the treatment arm. There are no existing standards for this balance of joint outcomes; however, this design will allow us to evaluate efficacy within a reasonable boundary of safety.

Conceptually, the power associated with the safety component of the composite test is the probability of achieving one of the two criteria defined above. To evaluate this probability, we simulated study outcomes for 10,000 studies with sample sizes in the range of 350 to 400 subjects per treatment arm. The prevalence of survival without BPD was assumed to be 25% to 35% on the control arm with the hydrocortisone arm providing an improvement of 10%. The probability of survival without NDI on the control arm was assumed to be 42% based on available NRN data with an assumed improvement of 1% to 3% on the hydrocortisone arm based on data available from the literature. The correlation between death or BPD at 36 weeks and death or NDI at 18 to 22 months was assumed to be in the range of 0.4 to 0.5 based on available data from the NRN GDB.

The results indicate that if the improvement in the death or NDI outcome is only 1% on the hydrocortisone arm, then the probability of achieving a “success” on the safety outcome is 0.68 to 0.71 if success requires the lower limit of a 95% confidence interval on the risk benefit ratio to exceed 4, but 0.80 to 0.81 if we only require that the point estimate achieve 4. However, if the true benefit of the hydrocortisone treatment on death or NDI is at least 3% then the probability of achieving a “success” on the safety outcome is 0.83 to 0.86 if success requires the lower limit of a 95% confidence interval on the risk benefit ratio to exceed 4, and 0.90 to 0.93 if we only require that the point estimate achieve 4.

(d) Secondary outcomes and analysis: After analysis of unadjusted risk differences, other factors known to affect neurodevelopmental outcomes (such as gender, maternal education and dexamethasone exposure) will be entered into a multivariable analysis of outcome using the robust Poisson regression models described for the primary efficacy outcome for each of the binary secondary outcomes defined below. Secondary outcomes will include:

(i) Standard NRN measures of morbidity and growth as collected for the GDB at 36 weeks postmenstrual age and at 18 – 22 months corrected age. We will include ROP incidence and severity as a secondary outcome of specific interest, as there has been conflicting information regarding the relationship of dexamethasone to ROP. We will also include a questionnaire to assess breathing outcomes at 18 – 22 months of age using forms developed for the SUPPORT study.

(ii) Successful extubation during the intervention period.

(iii) Use of open-label dexamethasone

(iii) Respiratory status at 40 weeks estimated gestational age. Assessment of respiratory outcomes at this age has been reported to more accurately predict long term respiratory outcomes (personal communication Michele Walsh). This assessment will not include a room air challenge, as repeating the physiologic testing would be burdensome and the 40 week status is not the primary study outcome.

(e) Concomitant medications will be recorded, in particular diuretics, bronchodilators, antibiotics, iNO, caffeine and Vitamin A.

(f) Safety monitoring:

(i) Adverse events to be monitored include those previously associated with glucocorticoid therapy, such as hyperglycemia, hypertension and gastrointestinal perforation (8, 9, 34). Any infant who develops new, sustained hyperglycemia (>180mg/dl on at least two determinations at least 6 hours apart) or new, sustained hypertension (mean arterial pressure >95th percentile for age on four serial determinations over at least 12 hours (37)) may have study drug held or discontinued if, in the opinion of the attending neonatologist, there is no plausible alternative explanation for these findings (such as new thrombus). Delaying the study window to 14 days and excluding infants receiving indomethacin/ibuprofen in the preceding 48 hours should minimize the possibility for spontaneous GI perforation (34).

We will also specifically monitor for signs of adrenal insufficiency after study drug is discontinued, including hypotension and oliguria, as well as hyponatremia and hyperkalemia on clinically obtained electrolyte specimens. Previous studies of hydrocortisone at various doses have not reported these signs after discontinuation of a tapering course of the drug (18, 38-40 and personal communication N. Parikh (22)). Patients who develop hypotension and oliguria (shock) significant enough in the judgment of the attending neonatologist to require support with volume and/or vasopressors may have a clinically indicated blood sample drawn for cortisol and may be restarted on hydrocortisone or other therapy for shock at the discretion of the clinical attending. Open-label hydrocortisone should be given at a test dose of 1mg/kg, with continuation of the therapy based on clinical response to the first dose.

(ii) Reporting of adverse events: In this high risk population, serious adverse events, such as nosocomial sepsis, respiratory deterioration or pneumonia, and death, occur with high frequency. Previously documented incidence and center variations in incidence of these serious adverse events in a similar population (infants receiving mechanical ventilation at 14 days in the NRN benchmarking study) will be prepared and forwarded to the DSMC before patient enrollment begins.

The DCC will compare the incidence of serious adverse events (nosocomial sepsis, intestinal perforation, respiratory deterioration or pneumonia, or death) between the two treatment arms after every 100th infant reaches status (death, discharge, transfer or 120 days of age). Since 800 infants are to be enrolled in this trial, this will involve 8 interim looks, giving a Pocock boundary of ±2.555, with a corresponding significance level of 0.011 (Pocock, 1977). Such reports will be forwarded to the Chair of the DSMC when there is any suggestion of a trend towards a difference between the two treatment arms emerging. They will also be made available to the full DSMC at their scheduled meetings after 50% and 75% of the trial infants have been evaluated for the survival without BPD efficacy outcome at 36 weeks postmenstrual age (see Section on futility monitoring on page 16 below). Serious adverse events to be reported to NICHD within 24 hours include death or intestinal perforation with an assessment as best as possible as to the cause of the perforation (NEC, spontaneous, other).

(9) Available population (based on GDB data, 06-07):

To create a conservative estimate of the population available for this study, we obtained from Dr. Das the number of babies 401 – 1500g birth weight who had received ≥14 days on mechanical ventilation during the 1st 28 days in the calendar years 2006 and 2007:

1242 (401 – 1000g), 214 (1001 – 1500g)	total = 1456, or 728/year
- If ¾ of those meet eligibility	= 546/year
- Consent rate at 60% (direct benefit study*)	= 328/year
- Total over a 3 year enrollment	= 983
- For a sample size of 800	= 30 months

(*glutamine consent rate was 70%)

Competing studies: The primary outcome for inositol is ROP, rather than BPD. Hydrocortisone treatment has not been shown to have effects on ROP. While inositol may have secondary effects on respiratory morbidities, these studies may be compatible. It appears from NRN conversations with the FDA that subjects may participate in more than one IND trial; therefore, this study would not conflict with probiotics.

RISKS, BENEFITS, POTENTIAL PROBLEMS

Risks:

(1) Spontaneous gastrointestinal perforation is primarily observed before 14 days postnatal age in infants receiving concurrent indomethacin/ibuprofen treatment (34, 38, 39). Risk will be minimized by starting the study after 14 days postnatal age, and by excluding infants who have received indomethacin/ibuprofen therapy within 48 hours prior to study entry.

(2) Short-term adverse effects of glucocorticoids, such as hyperglycemia, hypertension, growth failure. These effects have not been seen in RCTs of hydrocortisone vs. placebo (38-40); the plan for monitoring and addressing possible hyperglycemia and hypertension is outlined above.

(3) Adverse neurodevelopmental effects: This has not been reported as a consequence of hydrocortisone administration either in cohort studies or in RCTs. We will assess outcomes at 18 – 22 months corrected age and monitor for any such effects.

Benefits:

If hydrocortisone proves effective in reducing time on mechanical ventilation, this therapy will benefit these individual patients by decreasing the ongoing noxious effects of an indwelling endotracheal tube, suctioning and positive pressure ventilation. If hydrocortisone proves effective in decreasing the incidence of moderate or severe BPD through its effect on decreasing mechanical ventilation and/or its anti-inflammatory effect, it will benefit these individual patients by decreasing oxygen exposure and decreasing BPD as a factor associated with adverse neurodevelopmental outcomes. Based on previous data, hydrocortisone treatment may be associated with improvement in neurodevelopmental outcomes (20).

Possible problems:

(1) Futility: hydrocortisone may not prove effective for extubation or reduction of BPD. When approximately 50% and 75% of patients have been evaluated for the survival without BPD efficacy outcome at 36 weeks postmenstrual age, RTI will provide the DSMC with the information required to evaluate whether to stop the trial for futility. The analyses will include formal computation of conditional power for the primary outcome based on the observed data and the effect size and nuisance parameters used for the original study design and for key secondary parameters (including successful extubation during study drug administration, respiratory status at 40 weeks EGA, days on mechanical ventilation, days on positive pressure, days on oxygen, days exposed to open-label dexamethasone or other steroids (including inhaled steroids), length of stay, medications at discharge, growth parameters at 36 weeks EGA, nosocomial sepsis after study entry, necrotizing enterocolitis after study entry and retinopathy of prematurity) based on observed effect sizes and nuisance parameters. Should these analyses show a conditional power for the primary outcome of 0.1 or less and provide the DSMC with little evidence of a benefit for any of the secondary parameters, the DSMC will consider stopping enrollment in the trial for futility. Details for these futility analyses will be included in the DSMC monitoring plan. Patients who have been enrolled in the study will be followed through the 18 – 22 month evaluation.

(2) Slow patient enrollment: A timeline for enrollment is proposed, above, based on eligible patients anticipated at the NRN centers. This will be monitored, and if patient enrollment does not meet expected, the reasons for the problem will be analyzed and the potential for study completion assessed. Study forms will be constructed to include reasons for lack of enrollment, to assist in this process.

BUDGET (not including RTI costs) based on 800 patients

Pharmacy cost (avg): $2000/center set-up fee + $500/year x 3 years = $ 56,000

	Per patient (28 doses x $20/dose) =	$448,000*
Coordinator time:	10 hours/patient @ $35/hr ($350/pt)	$280,000
Follow up (est. deaths 15%, follow up rate 90% = 612):		
	$400 GDB babies, $700/pt otherwise (est.2/3)	$367,200
Estimated total:		$1,151,200

(*This may be lower, since all doses for each day can be prepared and dispensed at one time)

REFERENCES

1. Morley CJ, Davis PG, Doyle LW et al, COIN Trial Investigators. Nasal CPAP or intubation at birth for very preterm infants. N Engl J Med 2008; 358:700-8.
2. Walsh M, Laptook A, Kazzi SN, et al (National Institute of Child Health and Human Development Neonatal Research Network). A cluster-randomized trial of benchmarking and multimodal quality improvement to improve rates of survival free of bronchopulmonary dysplasia for infants with birth weights of less than 1250 grams. Pediatrics. 2007; 119:876-90.
3. Vohr BR, Wright LL, Poole WK, McDonald SA. Neurodevelopmental outcomes of extremely low birth weight infants <32 weeks' gestation between 1993 and 1998. Pediatrics. 2005; 116:635-43.
4. Walsh MC, Yao Q, Horbar JD, et al. Changes in the use of postnatal steroids for bronchopulmonary dysplasia in 3 large neonatal networks. Pediatrics 2006; 118:e1328-35.
5. Shinwell ES, Lerner-Geva L, Lusky A, Reichman B. Less postnatal steroids, more bronchopulmonary dysplasia: a population-based study in very low birthweight infants. Arch Dis Child Fetal Neonatal Ed. 2007; 92:F30-3.
6. Kobaly K, Schluchter M, Minich N, et al. Outcomes of extremely low birth weight (<1 kg) and extremely low gestational age (<28 weeks) infants with bronchopulmonary dysplasia: effects of practice changes in 2000 to 2003. Pediatrics. 2008; 121:73-81.
7. Yoder BA, Harrison M, Clark RH. Time-related changes in steroid use and bronchopulmonary dysplasia in preterm infants. Pediatrics. 2009; 124:673-9.
8. Halliday HL, Ehrenkranz RA, Doyle LW. Early (< 8 days) postnatal corticosteroids for preventing chronic lung disease in preterm infants. Cochrane Database Syst Rev. 2009 Jan 21; (1):CD001146
9. Halliday HL, Ehrenkranz RA, Doyle LW. Late (>7 days) postnatal corticosteroids for chronic lung disease in preterm infants. Cochrane Database Syst Rev. 2009 Jan 21; (1):CD001145.
10. Barrington KJ. The adverse neuro-developmental effects of postnatal steroids in the preterm infant: a systematic review of RCTs. BMC Pediatr 2001; 1:1.
11. Yeh TF, Lin YJ, Lin HC, et al. Outcomes at school age after postnatal dexamethasone therapy for lung disease of prematurity. N Engl J Med 2004; 350:1304-13.
12. American Academy of Pediatrics Committee on Fetus and Newborn, Canadian Paediatric Society Fetus and Newborn Committee. Postnatal corticosteroids to treat or prevent chronic lung disease in preterm infants. Pediatrics 2002; 109:330-8.
13. Doyle LW, Halliday HL, Ehrenkranz RA, et al. Impact of postnatal systemic corticosteroids on mortality and cerebral palsy in preterm infants: effect modification by risk for chronic lung disease. Pediatrics. 2005; 115:655-61.
14. McEvoy C, Bowling S, Williamson K, McGaw P, Durand M. Randomized, double-blinded trial of low-dose dexamethasone: II. Functional residual capacity and pulmonary outcome in very low birth weight infants at risk for bronchopulmonary dysplasia. Pediatr Pulmonol. 200; 38:55-63.

15. Doyle LW, Davis PG, Morley CJ, et al. (DART Study Investigators). Low-dose dexamethasone facilitates extubation among chronically ventilator-dependent infants: a multicenter, international, randomized, controlled trial. Pediatrics. 2006; 117:75-83.
16. Doyle LW, Davis PG, Morley CJ, et al (DART Study Investigators). Outcome at 2 years of age of infants from the DART study: a multicenter, international, randomized, controlled trial of low-dose dexamethasone. Pediatrics. 2007; 119:716-21.
17. Stark AR, Carlo W, Vohr BR, et al, *NICHD Neonatal Research Network.* Neurodevelopmental outcome and growth at 18 – 22 months. Pediatr Res 2001; 49:388A (abstract).
18. van der Heide-Jalving M, Kamphuis PJ, van der Laan MJ, et al. Short- and long-term effects of neonatal glucocorticoid therapy: is hydrocortisone an alternative to dexamethasone? Acta Paediatr 2003; 92:827-35.
19. Peltoniemi OM, Lano A, Puosi R, Yliherva A, Bonsante F, Kari MA, Hallman M. Trial of early neonatal hydrocortisone: two-year follow-up. Neonatology. 2008; 95:240-247.
20. Watterberg KL, Shaffer ML, Mishefske MJ, et al. Growth and neurodevelopmental outcomes after early low-dose hydrocortisone treatment in extremely low birth weight infants. Pediatrics 2007; 120: 40 - 48.
21. Rademaker KJ, Uiterwaal CS, Groenendaal F, et al. Neonatal hydrocortisone treatment: neurodevelopmental outcome and MRI at school age in preterm-born children. J Pediatr. 2007; 150:351-7.
22. Parikh NA, Lasky R, Tyson J, Kennedy K, Zhang Y, Yu X. Does low dose hydrocortisone improve volumetric MRI measures of cerebral growth in extremely low birth weight infants at high risk for bronchopulmonary dysplasia?: a randomized trial. E-PAS2009:3212.2
23. McEwen BS. The brain is an important target of adrenal steroid actions: a comparison of synthetic and natural steroids. Ann NY Acad Sci 1997; 823: 201-13.
24. De Kloet RE, Vreugdenhil E, Oitzl MS, Joels M. Brain corticosteroid receptor balance in health and disease. Endocrine Rev 1998; 19:269-301.
25. Sloviter RS, Sollas AL, Neubort S. Hippocampal dentate granule cell degeneration after adrenalectomy in the rat is not reversed by dexamethasone. Brain Res 1995; 682: 227-30.
26. Hassan AHS, von Rosenstiel P, Patchev VK, et al. Exacerbation of apoptosis in the dentate gyrus of the aged rat by dexamethasone and the protective role of corticosterone. Exp Neurol 1996; 140:43-52.
27. Isaacs EB, Lucas A, Chong WK, et al. Hippocampal volume and everyday memory in children of very low birth weight. Pediatr Res 2000; 47: 713-720.
28. Giménez M, Junqué C, Narberhaus A, et al. Hippocampal gray matter reduction associates with memory deficits in adolescents with history of prematurity. Neuroimage 2004; 23:869-77.
29. Nosarti C, Al-Asady MH, Frangou S, Stewart AL, Rifkin L, Murray RM. Adolescents who were born very preterm have decreased brain volumes. Brain. 2002; 125:1616-23.
30. Murphy BP, Inder TE, Huppi PS et al. Impaired cerebral cortical gray matter growth after treatment with dexamethasone for neonatal chronic lung disease. Pediatrics 2001; 107: 217-21.
31. Parikh NA, Lasky RE, Kennedy KA, et al. Postnatal dexamethasone therapy and cerebral tissue volumes in extremely low birth weight infants. Pediatrics. 2007; 119:265-72.

32. Thompson DK, Wood SJ, Doyle LW, et al. Neonate hippocampal volumes: prematurity, perinatal predictors, and 2-year outcome. Ann Neurol 2008; 63:642-51.
33. Rademaker KJ, Rijpert M, Uiterwaal CS, et al. Neonatal hydrocortisone treatment related to 1H-MRS of the hippocampus and short-term memory at school age in preterm born children. Pediatr Res. 2006; 59:309-13.
34. Attridge JT, Clark R, Walker MW, Gordon PV. New insights into spontaneous intestinal perforation using a national data set: (2) two populations of patients with perforations. J Perinatol 2006; 26:185-8.
35. Finer NN, Powers RJ, Ou CH, Durand D, Wirtschafter D, Gould JB; California Perinatal Quality Care Collaborative Executive Committee. Prospective evaluation of postnatal steroid administration: a 1-year experience from the California Perinatal Quality Care Collaborative. Pediatrics. 2006 Mar;117(3):704-13.
36. Yeager MP, Rassias AJ, Fillinger MP, Discipio AW, Gloor KE, Gregory JA, Guyre PM. Cortisol antiinflammatory effects are maximal at postoperative plasma concentrations. Crit Care Med. 2005; 33:1507-12.
37. Pejovic B, Peco-Antic A, Marinkovic-Eric J. Blood pressure in non-critically ill preterm and full-term neonates. Pediatr Nephrol. 2007; 22:249-57.
38. Watterberg KL, Gerdes JS, Cole CH et al. Prophylaxis of early adrenal insufficiency to prevent bronchopulmonary dysplasia: a multicenter trial. Pediatrics 2004; 114:1649-57.
39. Peltoniemi O, Kari MA, Heinonen K et al. Pretreatment cortisol values may predict responses to hydrocortisone administration for the prevention of bronchopulmonary dysplasia in high-risk infants. J Pediatr 2005; 146: 632-7.
40. Bonsante F, Latorre G, Iacobelli S, Forziati V, Laforgia N, Esposito L, Mautone A. Early low-dose hydrocortisone in very preterm infants: a randomized, placebo-controlled trial. Neonatology. 2007;91:217-21.

A RANDOMIZED CONTROLLED TRIAL OF THE EFFECT OF HYDROCORTISONE ON SURVIVAL WITHOUT BRONCHOPULMONARY DYSPLASIA AND ON NEURODEVELOPMENTAL OUTCOMES AT 22 - 26 MONTHS OF AGE IN INTUBATED INFANTS <30 WEEKS GESTATIONAL AGE

Kristi Watterberg, University of New Mexico
Michele Walsh, Case Western Reserve University
Kathleen Kennedy, University of Texas, Houston
Ron Goldberg, Duke University
Matthew Laughon, University of North Carolina, Chapel Hill
Carl D'Angio, University of Rochester
Abhik Das, RTI
Dennis Wallace, RTI
Conra Lacy, University of New Mexico
Georgia McDavid, University of Texas, Houston
Nancy Morgan, RPh

Version 3.0
Final: May 20, 2011
Revised: May 7, 2012
Revised: August 27, 2013

ABSTRACT:

Bronchopulmonary dysplasia (BPD) remains a leading morbidity of the extremely preterm infant, and prolonged mechanical ventilation is associated with increased risk for BPD. Dexamethasone has been used previously to facilitate extubation and decrease the incidence of BPD; however, due to adverse effects on neurodevelopmental outcomes, the use of this drug has decreased. One cohort study suggests that hydrocortisone (HC) may facilitate extubation. HC has thus far not been associated with adverse neurodevelopmental outcomes in either cohort studies or randomized controlled trials. A recent meta-analysis of postnatal corticosteroid therapy begun after the first week of life suggested that “late therapy may reduce neonatal mortality without significantly increasing the risk of adverse long-term neurodevelopmental outcomes,” although the methodological quality of some of the follow-up was acknowledged to be limited.

We propose a randomized controlled trial to study the efficacy and safety of a 10-day tapering course of hydrocortisone treatment for infants <30 weeks estimated gestational age at birth who remain intubated at 14 - 28 days postnatal age. Based on previous Network data these criteria define a population with a risk of death or BPD at 36 weeks postmenstrual age of approximately 65 – 75%. The primary outcome for this study will incorporate both (1) survival without moderate to severe BPD by Network physiologic definition and (2) survival without moderate or severe NDI at 22 - 26 months corrected age. Therefore, the results of this study will be reported only when follow-up data are available unless (1) the trial is stopped early by the DSMC because of strong evidence of benefit or harm, or (2) at the time all subjects have completed treatment the DCC finds a substantial survival benefit favoring hydrocortisone ($p<0.001$). Individual study assignment will remain masked until the follow-up is completed. Secondary outcomes will include short term measures such as respiratory morbidities and growth at 36 weeks postmenstrual age and long term measures including growth and other outcomes at 22 - 26 months corrected age.

STATEMENT OF THE PROBLEM

Bronchopulmonary dysplasia remains a leading morbidity of the extremely preterm infant, resistant to therapeutic interventions and associated with adverse neurodevelopmental outcomes (1-3). Most studies indicate that its rate of occurrence has not decreased in recent years (4), and several reports suggest that the incidence and/or severity of the disease may be increasing as postnatal glucocorticoid use has decreased (5-7). Although dexamethasone can facilitate extubation and decrease the incidence of BPD (8, 9), such treatment can be associated with increased risk of short- and long-term complications including impaired growth and neurodevelopmental delay (10 – 12). However, because BPD also is associated with increased risk for death and adverse neurodevelopmental outcomes, meta-analyses have suggested that in populations at high risk for death or BPD even dexamethasone therapy may be associated with net benefit (9, 13). Lower doses of dexamethasone may have equal pulmonary benefit with less toxicity (14 – 17); however, because it has a very long biologic half-life, suppresses endogenous cortisol production and disrupts the balance between mineralocorticoid and glucocorticoid in the brain (see below), dexamethasone may still be an inferior glucocorticoid choice for premature infants. Establishment of an alternative therapeutic agent to facilitate extubation may improve respiratory outcomes for these extremely preterm infants at very high risk for BPD, without the adverse effects previously seen with dexamethasone therapy.

HYPOTHESIS

Compared to placebo, hydrocortisone treatment of infants <30 weeks estimated gestational age at birth who remain on mechanical ventilation at 14 – 28 days postnatal age will significantly improve their rate of survival without moderate or severe BPD and will be associated with improvement in survival without moderate or severe neurodevelopmental impairment at 22 - 26months corrected age.

SPECIFIC AIMS

- To determine whether a 10 day course of hydrocortisone results in a significant increase in survival without moderate or severe BPD;
- To determine whether a 10 day course of hydrocortisone results in a significant increase in the number of infants successfully extubated during treatment;
- To determine whether this treatment affects other short-term outcomes including respiratory morbidities, growth and length of stay;
- To determine whether this treatment affects survival without moderate or severe NDI at 22 - 26months corrected age; and,
- To determine whether this treatment affects other outcomes, such as growth and pulmonary morbidity, at 22 -26 months

RATIONALE/JUSTIFICATION/PREVIOUS STUDIES

Glucocorticoid therapy to prevent or treat BPD began with high doses of dexamethasone, a powerful synthetic glucocorticoid with a myriad of predictable adverse effects on short and

long term outcomes, including growth and neurodevelopment (8-12). Lower doses may have equal pulmonary benefit with less toxicity (14-17); however, because it has a very long biologic half-life, suppresses endogenous cortisol production and disrupts the balance between mineralocorticoid and glucocorticoid in the brain (see below), dexamethasone may still be an inferior glucocorticoid choice for premature infants. One cohort study has reported that hydrocortisone may be efficacious in facilitating extubation in preterm infants (18).

Data regarding the effect of neonatal hydrocortisone treatment on neurodevelopmental outcomes are encouraging. A meta-analysis of three RCTs evaluating 333 infants treated for the first two weeks of life with 1-2 mg/kg/day of hydrocortisone showed no difference at 18 – 22 months corrected age in the incidence of cerebral palsy (12% in each group) and a non-significant *increase* in survival without NDI (54% HC vs. 49% placebo) (19). The largest of the included studies (252 infants) showed suggestions of benefit, with a significantly lower incidence of Bayley-II MDI <70 and increased “awareness of object permanence”, an early measure of executive function and pre-frontal cortical development (20). At school age (7 – 8 years of age), outcome data from a cohort study comparing 62 infants treated with a 22 day course of hydrocortisone for BPD (median start 19d, initial dose 5mg/kg/day) to 164 non-treated infants who were larger, more mature and less sick showed no evidence of adverse functional or structural (MRI) effects (21). A recent RCT designed to evaluate the effects of hydrocortisone therapy for BPD on brain growth showed that hydrocortisone treatment (a tapering dose over 7 days, starting at 3mg/kg/day, total 17mg/kg) had no adverse effects on brain volumes on MRI at 38 weeks postmenstrual age (n=48, entry from 10 – 21 days postnatal age) (22).

Differences in the actions of dexamethasone and hydrocortisone in the brain may contribute to the observed differences in neurodevelopmental outcomes. Two types of corticosteroid receptors are found in the brain: mineralocorticoid and glucocorticoid. Under basal conditions, cortisol binds preferentially to mineralocorticoid receptors in the brain; at times of higher stress, cortisol also binds to glucocorticoid receptors (23, 24). Dexamethasone is a powerful synthetic glucocorticoid with no mineralocorticoid activity and a very long biologic half-life. It suppresses endogenous cortisol production, leaving mineralocorticoid receptors unoccupied, thereby creating a “chemical adrenalectomy” (23, 24). A lack of cortisol binding to mineralocorticoid receptors has been shown in animal studies to result in neuronal apoptosis. This apoptosis occurs whether the lack of cortisol binding is the result of a cortisol deficiency produced by adrenalectomy or the result of dexamethasone administration (25, 26). Further, concurrent administration of corticosterone (the cortisol equivalent in rats) with dexamethasone has been shown to protect against dexamethasone-induced apoptosis (25, 26).

Corticosteroid receptors are found in high density in the hippocampus, an area of the brain critical for learning and memory (26-29). Individuals born preterm have reductions in hippocampal volume compared to term (27-29), and hippocampal gray matter reduction has been associated with memory deficits in adolescents born prematurely (28). In small cohort studies, preterm infants treated with dexamethasone have been found to have decreased grey matter and/or hippocampal volume compared to untreated controls (30-32); however, those treated with hydrocortisone have shown no reduction in volume or increase in lesions vs. untreated controls (21, 33).

Thus, there is both biologically plausible basis for preferring hydrocortisone over dexamethasone and clinical evidence in preterm infants that hydrocortisone does not impair long term neurodevelopmental outcomes whereas dexamethasone can do so. In addition, there is

preliminary evidence from a small cohort study that hydrocortisone is equally efficacious as dexamethasone in facilitating extubation in preterm infants. Therefore, we propose a randomized, masked, placebo-controlled trial to study (1) the efficacy of hydrocortisone in facilitating extubation and thereby increasing survival without moderate or severe BPD and (2) the safety of HC for this use, by assessing the effect of hydrocortisone on survival without moderate or severe NDI at 22 - 26months adjusted age.

METHODS/PROCEDURES

(1) Study design: Randomized, masked placebo-controlled trial.

(2) Study population: Patients eligible for this study will be infants between 14 – 28 postnatal days who:

(a) are <30 weeks estimated gestational age, to be randomized in two strata: $\leq 26^6$ and $27^0 – 29^6$ weeks);

(b) were inborn at an NRN site or were admitted to an NRN site ≤ 72 hours postnatal age;

(c) have received ≥ 7days of mechanical ventilation;

(d) are receiving mechanical ventilation through an endotracheal tube .

This population is at very high risk for death or moderate to severe BPD, based on previous NRN data (approximately 65 – 75%, see data below), and is a population for whom the most recent Cochrane review suggests it is reasonable to consider corticosteroid therapy (9). Deferring study entry until 14 days postnatal age will avoid the period of highest risk for spontaneous intestinal perforation (34).

Data provided by Dr. Das for the two year period 2006 – 2007, using the physiologic definition of moderate to severe BPD, i.e., supplemental oxygen and/or positive pressure ventilation at 36 weeks postmenstrual age showed that:

Of infants who received ≥ 7 days of mechanical ventilation in the first 14 days, the percentage surviving without BPD was:

```
 401-999g BW: 368/1482(24.83%)
1000-1500 BW: 166/399 (41.80%)
Total (401-1500 BW): 534/1881(28.4%)
```

Of infants who received ≥ 7 days of ventilation in the first 28 days, the percentage surviving without BPD was:

```
 401-999g BW: 457/1528(29.91%)
1000-1500 BW: 229/490 (46.73%)
Total (401-1500 BW): 686/2018 (33.99%)
```

Of infants who received ≥ 14 days of ventilation in the first 28 days, the percentage surviving without BPD was:

```
  401-999g BW: 292/1249(23.38%)
 1000-1500 BW: 57/222 (25.68%)
 Total (401-1500 BW): 349/1471(23.73%)
```

In addition, unpublished data from the SUPPORT trial showed that of the 526 infants who were on mechanical ventilation at 14 days, 372 (71%) died or developed BPD.

We will include infants who have failed extubation because of apnea, because they too are at risk for BPD from prolonged intubation. HC may promote successful extubation in these infants by decreasing ongoing lung inflammation and capillary leak and improving FRC.

(3) Exclusions:

(a) Major congenital anomalies

(b) Decision to limit support

(c) Indomethacin or ibuprofen treatment within 48 hours of study drug

(d) Previous corticosteroid treatment for BPD

(e) Hydrocortisone treatment for hypotension in the first week of life is common (35) and will not be an exclusion; however, infants will be excluded if they have received hydrocortisone or any other systemic glucocorticoid:

(i) for ≥ 14 cumulative days OR

(ii) within 7 days of study entry.

(f) Enrolled in a conflicting clinical trial

(4) Study procedures

(a) Ascertainment of study eligibility: Prior to the 14th postnatal day, the clinical care team will be asked whether they plan to extubate a potentially eligible infant within the next 24 – 48 hours. If so, approach for parental consent will be deferred for that time period. If the infant is not extubated by the 14th postnatal day, or if he/she fails extubation within the study window, AND if the attending physician agrees to the patient's entry into the study, then the parents will be approached for consent.

(b) Randomization procedures:

i. Infants will be randomized 1:1 to the two arms using a stratified, permuted block design with strata for gestational age as described above within center.

ii. After parental consent is obtained, the NRN coordinator at each center will randomize subjects by telephoning the Data Coordinating Center at RTI International, Research Triangle Park, NC; the randomization system will be available 24 hours per day, 7 days per week.

iii. Pharmacy personnel will prepare and dispense study drug in accordance with procedures described in the study manual. HC and normal saline placebo are indistinguishable in appearance.

(c) Therapeutic intervention: The infant will be randomized to saline placebo or hydrocortisone sodium succinate for intravenous administration (unpreserved, Solu-Cortef plain, Pfizer®, reconstituted with unpreserved normal saline to avoid exposure to the benzyl alcohol contained in preserved diluents), to be administered either intravenously or orally if no intravenous line is available (route of delivery will be recorded on drug administration forms), at the same dose, and tapered as follows:

4mg/kg/day ÷ q 6 hours x 2 days, then
2mg/kg/day ÷ q 6 hours x 3 days; then
1mg/kg/day ÷ q 12 hours x 3 days; then
0.5mg/kg/d as a single dose x 2 days

Rationale for dosing: A dose of 4mg/kg/day is intended to reach anti-inflammatory plasma concentrations (36), and is similar to the starting hydrocortisone dose in the previously reported cohort study (5mg/kg/day) (18) and the dose of dexamethasone given in the DART study (starting at 0.15mg/kg, ≅ 4 – 6mg/kg/day hydrocortisone) (15). The length of therapy is shorter than the previous hydrocortisone cohort study (18), but equal to the DART study, consistent with an overall goal of giving lower doses of glucocorticoid for shorter periods of time (9).

Need to taper study drug if parents withdraw consent during therapy: Sudden withdrawal of glucocorticoid therapy may precipitate acute adrenal insufficiency; therefore, language will be included in the consent form as follows: ***"If you should decide to withdraw your child from the study during the study drug administration period, for reasons of safety, your child will have the study drug gradually decreased until off"***. If parents withdraw consent for their infant to participate in this trial during the period of study drug administration, the study will NOT be unblinded, and the following taper protocol will be utilized:

- If up to 8 doses have been given (4mg/kg/day dosing level): drop to the next dose level x 2 days, then subsequent dose x 3 days, then lowest dose for 2 days.

- If 9 - 12 doses have been given (first day of the 2mg/kg/day dose): stay at this dose for one more day (total of 8 doses at this dosing level), then drop to the subsequent dosing level (1mg/kg/day) for 3 days, then lowest dose for 2 days.

- If 13 - 16 doses have been given (day 2 of the 2mg/kg/day dose): stay at this dose through dose #16, then drop to the subsequent dosing level (1mg/kg/day) for 3 days, then lowest dose for 2 days.

- If 17 - 20 doses have been given (day 3 of the 2mg/kg/day dose): drop to next dosing level (1mg/kg/day) for 3 days, then lowest dose for 2 days.

- From dose 21 on, taper as described in the study protocol.

If parents refuse to have the study drug weaned by this accelerated taper even after discussion with investigator or clinical attending physician, the blind may be broken to determine the best course of action.

<u>(d) Extubation:</u> Infants at this age may not have blood gases obtained regularly. Therefore, extubation criteria include information available without a blood gas, and were targeted toward the lower end of the criteria in the SUPPORT trial. Extubation must be attempted within 72 hours of starting study drug and within 24 hours after meeting the following criteria: the FiO2 is <0.40 to maintain a saturation of 88%, the mean airway pressure is < 8, and the infant is hemodynamically stable (as defined for the SUPPORT trial: "clinically acceptable blood pressure and perfusion in the opinion of the clinical team"). The infant may be extubated from higher settings at the discretion of the attending physician. If the infant does not meet these criteria within 72 hours, he/she will be reassessed every 24 hours during the intervention period and extubation will be attempted within 24 hours of those criteria being met. If the infant **is intentionally extubated per study criteria** and subsequently re-intubated, further attempts at extubation will be at the discretion of the clinical care team. [**<u>Note</u>**: inadvertent extubation and subsequent re-intubation are not included in this statement. Such an infant will still be eligible for extubation per study protocol.]

<u>(e) Concurrent therapy with indomethacin/ibuprofen:</u> As stated above, infants who have received indomethacin or ibuprofen within 48 hours are not eligible for the study at that time. Spontaneous gastrointestinal perforation primarily occurs during the first 14 days of postnatal life; therefore, these infants should be at low risk for this event (34). However, to minimize any remaining risk, if a patient in this study is determined by the clinical care team to have developed a PDA during the intervention period which <u>must</u> be treated before study drug is completed, the attending physician <u>may</u> choose to stop the study drug prior to treatment with indomethacin or ibuprofen. If upon discontinuing study drug the patient becomes hypotensive and oliguric with an electrolyte profile consistent with adrenal insufficiency, the attending physician <u>may</u> choose to institute open-label hydrocortisone treatment or other clinically-indicated therapy for the hypotension.

(f) Clinical deterioration during study drug weaning: In this high-risk population, it is anticipated that some infants will experience clinical deterioration during or immediately following study drug therapy for unrelated causes, such as nosocomial sepsis, NEC or pneumonia. Although we do not anticipate that infants will experience clinical deterioration due to adrenal suppression during this short course of hydrocortisone, we will monitor for development of clinical signs of adrenal insufficiency during and immediately after treatment with study drug (see adverse event monitoring below). If an attending physician believes that the infant is experiencing hypotension with inadequate perfusion and oliguria (i.e., shock) due to adrenal insufficiency, a clinically-indicated blood specimen may be drawn for cortisol and the attending physician may institute therapies that he/she feels are clinically necessary for the care of the infant. Study drug should not be stopped for this occurrence. These events will be recorded on a study form created specifically for this study (to include above factors and attending physician judgment regarding the etiology of the event), and the event rate for such clinical deterioration in each group will be monitored by RTI and forwarded to the DSMC with other regularly transmitted study data.

(5) Post-extubation respiratory care

(a) Respiratory support: Each center currently cares for these infants with techniques they feel appropriate, whether CPAP, CPAP with IMV, high-flow nasal cannula or other. This study will not prescribe the technique to be used. Because this is an RCT and because enrollment will be stratified by center and study treatment will be masked, the center-specific and attending-specific techniques should be balanced in both treatment groups equally over the course of the trial and therefore not introduce bias.

(b) Open-label glucocorticoid therapy

(i) Infants who remain successfully extubated are not to be treated with open-label glucocorticoids as therapy for BPD. This will be a protocol violation.

(ii) Infants who are not extubated during the study treatment period or who are subsequently re-intubated may be treated with open-label dexamethasone after ≥ 4 days following the last dose of study drug. Open-label dexamethasone will be encouraged to be prescribed as described by Doyle (15). Further treatment with HC for prevention/treatment of BPD will be considered a protocol violation. The rationale for this plan is as follows: (1) HC has not been demonstrated in an RCT to be efficacious for extubation, whereas dexamethasone has (8, 9, 15); (2) this design will permit a true RCT of HC without open-label contamination of the placebo group. At the same time, we will not be denying treatment to the group of infants that meta-analysis suggests derive net benefit from treatment with dexamethasone (13)

(6) Definition of successful extubation: An infant who remains extubated for at least one week, including at least 3 days (72 hours) after the last dose of study medication, will be considered to be successfully extubated in response to this treatment.

(7) Laboratory specimens: none

(8) Analysis of study outcomes

(a) Study specifications and definitions

(i). Analysis will be by intention to treat.

(ii) Moderate or severe BPD is defined as the need for supplemental oxygen by NRN physiologic definition at 36 weeks estimated gestational age. This includes an oxygen withdrawal test for infants receiving <0.30FiO2, as described in the study manual of procedures. For this study, this determination will be made between 36° to 36^{6} weeks.

(iii) Infants discharged on oxygen prior to 36 weeks gestation will be evaluated at 36 weeks gestational age at a follow-up clinic visit, a home visit or by telephone if necessary.

(iv) Outcomes assessed at 22 - 26 months corrected age will be the standard NRN follow up assessments: demographics, medical history including neurosensory outcomes, physical and neurologic examination, neurodevelopmental and behavioral assessment following the NRN Follow-up protocol.

(v) "Severe neurodevelopmental impairment" will be defined as (1) a BSID-III cognitive score or BSID-III motor score more than 2 SD below the mean (<70); OR (2) a Gross Motor Function classification code of 4 – 5 (Level 2 or 3) indicating moderate to severe cerebral palsy; OR (3) severe to profound hearing impairment (no functional hearing with amplification); OR (4) bilateral blindness that cannot be corrected.

(vi) "Moderate neurodevelopmental impairment" will be defined as (1) BSID-III cognitive score or BSID-III motor score 1 – 2 SD below the mean (70 – 84); OR (2) a GMFCS code of 2 -3 (Possible Level 1 or Level 1) indicating no cerebral palsy or mild cerebral palsy; OR (3) moderate hearing impairment (can hear and follow directions with amplification);); OR (4) moderate vision impairment (limited vision with correction or unilateral blindness).

(vii) Because the primary outcome for this study includes evaluation at 22 - 26 months, earlier study outcomes will not be reported unless the DSMC stops the trial prematurely because of strong evidence of benefit or harm, or at the time all

subjects have completed treatment the DCC determines a substantial mortality benefit favoring hydrocortisone that is significant at $p<0.001$.

(b) Primary outcome measure and analytic approach: The primary outcome for this study will include both a measure of efficacy (improvement in survival without physiologically defined moderate to severe BPD) and safety (survival without moderate or severe NDI at 22 - 26 months corrected age). Because the primary study outcome will not be known until evaluation of the outcomes at 22 - 26 months corrected age, BPD outcome data will not be released until after this time.

We propose that if hydrocortisone treatment is efficacious in improving survival without moderate or severe BPD, this treatment will also result in an improvement in survival without NDI, since BPD is a risk factor for both mortality and adverse neurodevelopmental outcomes. Meta-analysis of early, low-dose hydrocortisone trials showed a 5% overall increase in survival without major NDI (19). The largest of those trials showed a significant improvement in the percentage of infants with MDI <70 (27% vs. 37%) and a 4 percentage point improvement in survival without NDI (0.68 (0.41–1.10)), even in the absence of a significant effect on BPD (20). No previous RCT or cohort study of hydrocortisone using 5mg/kg/day or less has shown adverse neurodevelopmental effects associated with its use (18-22). Meta-regression has shown that even dexamethasone, with its numerous short and long term adverse effects, provided net benefit for the outcome of death or cerebral palsy for infants with an *a priori* risk of BPD or death of >65% (13).

This study will provide a sequential evaluation of the composite hypothesis that (i) administration of hydrocortisone has efficacy benefit in that it reduces the risk of death or BPD at 36 weeks; and (ii) administration of hydrocortisone has an acceptable long-term safety profile with respect to death or NDI at 22-26 months. Specifically, the primary outcome for this study will include both a measure of efficacy (improvement in survival without BPD) and safety (survival without major NDI at 22 - 26months corrected age). This composite hypothesis will be evaluated sequentially. First, point and interval estimates of the adjusted relative risk obtained from a robust Poisson regression model controlling for center and gestational age will be used to test the hypothesis that infants treated with hydrocortisone had a lower risk of death or BPD at 36 weeks than infants treated with placebo. If this test of efficacy indicates benefit of the hydrocortisone arm then the safety of the treatment will be evaluated descriptively through an assessment of the risk to benefit ratio of the hydrocortisone treatment. Specifically, this safety outcome will be considered a "success" if either (1) the point estimate of risk of death/NDI is lower on the hydrocortisone arm than on the control arm, or (2) there is an increase in risk on the hydrocortisone arm, but the lower limit of a one-sided 95% confidence interval for the ratio of increased benefit for BPD to increased risk for NDI is greater than 4. In other words, for every additional 4 infants surviving without BPD, no more than 1 additional infant would experience death/NDI.

(c) Power calculations and sample size:

Consistent with the approach for evaluating the composite hypothesis defined above, the sample size calculations were conducted in two stages. First, standard power

analysis software was used to assess the sample size required to demonstrate a difference in death or BPD at 36 weeks on the two treatment arms. Second, simulation analyses were used to assess the probability of success for the safety outcome based on either of the two criteria defined above. The empirical probability of success for the descriptive analyses used to evaluate the safety outcome is analogous to the power of a formal hypothesis test.

As summarized in the subsections below, a sample size of 800 subjects will be adequate to provide the study with a power of 80% for the efficacy hypothesis and a probability of greater than 80% of a successful safety evaluation. With the population available (below), this sample size should allow completion of enrollment in less than 3 years, will give a clear answer regarding the efficacy of hydrocortisone for improving survival without moderate or severe BPD, and will allow a reasonable assessment of safety and potential benefit at 22 - 26 months corrected age.

(i) Efficacy:
Empirical studies of the effect of alternative analytic approaches for binary outcomes (e.g. tests of differences in proportions, logistic regression, Mantel-Haenszel tests, and Poisson regression models) indicate that unless the study has both substantial imbalance of sample sizes across strata and the substantial stratum-specific heterogeneity of treatment effect, power calculations for the various approaches can reasonably be approximated by standard tests for differences in proportions. Consequently, we used standard software to estimate sample sizes for a power of 0.80 under the assumption that the risk of death or BPD at 36 weeks on the control arm was in the range of 65% to 75% (or alternatively that the probability of survival without BPD was in the range of 25% to 35%). Considering a 10 percentage point difference in survival without BPD to be clinically significant, improving survival without BPD from approximately 25% in this population (historical NRN data) to 35% with a power of 0.80 would require a sample size of 658 infants. If the incidence of survival without BPD in the placebo group has improved over time to 35%, we can achieve a power of 0.80 to detect an increase to 45% with a sample size of 752. A sample size of 800 subjects will be adequate to assure that the study has a power of at least 80% to detect a 10 percentage point improvement in outcome regardless of the baseline incidence of survival without BPD, with a margin for a small loss in power if the treatment effect has modest heterogeneity across strata.

(ii) Safety: In view of the previous data suggesting possible benefit from hydrocortisone treatment, we anticipate that hydrocortisone treatment will be associated with improved survival without moderate or severe neurodevelopmental impairment (defined “NDI”) in this population. However, we do not anticipate demonstrating a statistically significant benefit with this sample size. Instead, we postulate based on previous data that hydrocortisone will provide a 3% improvement in survival without NDI (19,20), and will consider this outcome successful if either (1) the risk of death/NDI is lower on the hydrocortisone arm than on the control arm, or (2) there is an increase in

death/NDI in the hydrocortisone arm, but the lower limit of a one-sided 95% confidence interval for the ratio of increased benefit for BPD to increased risk for NDI is greater than 4. In other words, for every additional 4 infants surviving without moderate or severe BPD, we would have 95% confidence that no more than 1 additional infant would experience death/NDI. For example, if we achieve the successful outcome of an increase of 10% in survival without moderate or severe BPD, we would hypothesize also achieving an increase of 3% in survival without NDI, but would only define the treatment as a success if there were no more than a 2.5% increase in death or NDI in the treatment arm. There are no existing standards for this balance of joint outcomes; however, this design will allow us to evaluate efficacy within a reasonable boundary of safety.

Conceptually, the power associated with the safety component of the composite test is the probability of achieving one of the two criteria defined above. To evaluate this probability, we simulated study outcomes for 10,000 studies with sample sizes in the range of 350 to 400 subjects per treatment arm. The prevalence of survival without BPD was assumed to be 25% to 35% on the control arm with the hydrocortisone arm providing an improvement of 10%. The probability of survival without NDI on the control arm was assumed to be 42% based on available NRN data with an assumed improvement of 1% to 3% on the hydrocortisone arm based on data available from the literature. The correlation between death or BPD at 36 weeks and death or NDI at 18 to 22 months was assumed to be in the range of 0.4 to 0.5 based on available data from the NRN GDB.

The results indicate that if the improvement in the death or NDI outcome is only 1% on the hydrocortisone arm, then the probability of achieving a “success” on the safety outcome is 0.68 to 0.71 if success requires the lower limit of a 95% confidence interval on the risk benefit ratio to exceed 4, but 0.80 to 0.81 if we only require that the point estimate achieve 4. However, if the true benefit of the hydrocortisone treatment on death or NDI is at least 3% then the probability of achieving a “success” on the safety outcome is 0.83 to 0.86 if success requires the lower limit of a 95% confidence interval on the risk benefit ratio to exceed 4, and 0.90 to 0.93 if we only require that the point estimate achieve 4.

(d) Secondary outcomes and analysis: After analysis of unadjusted risk differences, other factors known to affect neurodevelopmental outcomes (such as gender, maternal education and dexamethasone exposure) will be entered into a multivariable analysis of outcome using the robust Poisson regression models described for the primary efficacy outcome for each of the binary secondary outcomes defined below. Secondary outcomes will include:

(i) Standard NRN measures of morbidity and growth as collected for the GDB at 36 weeks postmenstrual age and at 22 - 26 months corrected age. We will include ROP incidence and severity as a secondary outcome of specific interest, as there has been conflicting information regarding the relationship of dexamethasone to

ROP. We will also include a questionnaire to assess breathing outcomes at 22 - 26 months of age using forms developed for the SUPPORT study.
(ii) Successful extubation during the intervention period.

(iii) Use of open-label dexamethasone

(iii) Respiratory status at 40 weeks estimated gestational age. Assessment of respiratory outcomes at this age has been reported to more accurately predict long term respiratory outcomes (personal communication Michele Walsh). This assessment will not include a room air challenge, as repeating the physiologic testing would be burdensome and the 40 week status is not the primary study outcome.

(e) Concomitant medications will be recorded, in particular diuretics, bronchodilators, antibiotics, iNO, caffeine and Vitamin A.

(f) Safety monitoring:

(i) Adverse events to be monitored include those previously associated with glucocorticoid therapy, such as hyperglycemia, hypertension and gastrointestinal perforation (8, 9, 34). Any infant who develops new, sustained hyperglycemia (>180mg/dl on at least two determinations at least 6 hours apart) or new, sustained hypertension (mean arterial pressure >95th percentile for age on four serial determinations over at least 12 hours (37)) may have study drug held or discontinued if, in the opinion of the attending neonatologist, there is no plausible alternative explanation for these findings (such as new thrombus). Delaying the study window to 14 days and excluding infants receiving indomethacin/ibuprofen in the preceding 48 hours should minimize the possibility for spontaneous GI perforation (34).

We will also specifically monitor for signs of adrenal insufficiency after study drug is discontinued, including hypotension and oliguria, as well as hyponatremia and hyperkalemia on clinically obtained electrolyte specimens. Previous studies of hydrocortisone at various doses have not reported these signs after discontinuation of a tapering course of the drug (18, 38-40 and personal communication N. Parikh (22)). Patients who develop hypotension and oliguria (shock) significant enough in the judgment of the attending neonatologist to require support with volume and/or vasopressors may have a clinically indicated blood sample drawn for cortisol and may be restarted on hydrocortisone or other therapy for shock at the discretion of the clinical attending. Open-label hydrocortisone should be given at a test dose of 1mg/kg, with continuation of the therapy based on clinical response to the first dose.
(ii) Reporting of adverse events: In this high risk population, serious adverse events, such as nosocomial sepsis, respiratory deterioration or pneumonia, and death, occur with high frequency. Previously documented incidence and center variations in incidence of these serious adverse events in a similar population

(infants receiving mechanical ventilation at 14 days in the NRN benchmarking study) will be prepared and forwarded to the DSMC before patient enrollment begins.

The DCC will compare the incidence of serious adverse events (nosocomial sepsis, intestinal perforation, respiratory deterioration or pneumonia, or death) between the two treatment arms after every 100th infant reaches status (death, discharge, transfer or 120 days of age). Since 800 infants are to be enrolled in this trial, this will involve 8 interim looks, giving a Pocock boundary of ±2.555, with a corresponding significance level of 0.011 (Pocock, 1977). Such reports will be forwarded to the Chair of the DSMC when there is any suggestion of a trend towards a difference between the two treatment arms emerging. They will also be made available to the full DSMC at their scheduled meetings after 50% and 75% of the trial infants have been evaluated for the survival without BPD efficacy outcome at 36 weeks postmenstrual age (see Section on futility monitoring on page 16 below). Serious adverse events to be reported to NICHD within 24 hours include death or intestinal perforation with an assessment as best as possible as to the cause of the perforation (NEC, spontaneous, other).

(9) Available population (based on GDB data, 06-07):

To create a conservative estimate of the population available for this study, we obtained from Dr. Das the number of babies 401 – 1500g birth weight who had received ≥14 days on mechanical ventilation during the 1st 28 days in the calendar years 2006 and 2007:

1242 (401 – 1000g), 214 (1001 – 1500g)	total = 1456, or 728/year
- If ¾ of those meet eligibility	= 546/year
- Consent rate at 60% (direct benefit study*)	= 328/year
- Total over a 3 year enrollment	= 983
- For a sample size of 800	= 30 months

(*glutamine consent rate was 70%)

Competing studies: The primary outcome for inositol is ROP, rather than BPD. Hydrocortisone treatment has not been shown to have effects on ROP. While inositol may have secondary effects on respiratory morbidities, these studies may be compatible. It appears from NRN conversations with the FDA that subjects may participate in more than one IND trial; therefore, this study would not conflict with probiotics.

RISKS, BENEFITS, POTENTIAL PROBLEMS

Risks:

(1) Spontaneous gastrointestinal perforation is primarily observed before 14 days postnatal age in infants receiving concurrent indomethacin/ibuprofen treatment (34, 38, 39). Risk will be minimized by starting the study after 14 days postnatal age, and by excluding infants who have received indomethacin/ibuprofen therapy within 48 hours prior to study entry.

(2) Short-term adverse effects of glucocorticoids, such as hyperglycemia, hypertension, growth failure. These effects have not been seen in RCTs of hydrocortisone vs. placebo (38-40); the plan for monitoring and addressing possible hyperglycemia and hypertension is outlined above.

(3) Adverse neurodevelopmental effects: This has not been reported as a consequence of hydrocortisone administration either in cohort studies or in RCTs. We will assess outcomes at 22 - 26 months corrected age and monitor for any such effects.

Benefits:

If hydrocortisone proves effective in reducing time on mechanical ventilation, this therapy will benefit these individual patients by decreasing the ongoing noxious effects of an indwelling endotracheal tube, suctioning and positive pressure ventilation. If hydrocortisone proves effective in decreasing the incidence of moderate or severe BPD through its effect on decreasing mechanical ventilation and/or its anti-inflammatory effect, it will benefit these individual patients by decreasing oxygen exposure and decreasing BPD as a factor associated with adverse neurodevelopmental outcomes. Based on previous data, hydrocortisone treatment may be associated with improvement in neurodevelopmental outcomes (20).

Possible problems:

(1) Futility: hydrocortisone may not prove effective for extubation or reduction of BPD. When approximately 50% and 75% of patients have been evaluated for the survival without BPD efficacy outcome at 36 weeks postmenstrual age, RTI will provide the DSMC with the information required to evaluate whether to stop the trial for futility. The analyses will include formal computation of conditional power for the primary outcome based on the observed data and the effect size and nuisance parameters used for the original study design and for key secondary parameters (including successful extubation during study drug administration, respiratory status at 40 weeks EGA, days on mechanical ventilation, days on positive pressure, days on oxygen, days exposed to open-label dexamethasone or other steroids (including inhaled steroids), length of stay, medications at discharge, growth parameters at 36 weeks EGA, nosocomial sepsis after study entry, necrotizing enterocolitis after study entry and retinopathy of prematurity) based on observed effect sizes and nuisance parameters. Should these analyses show a conditional power for the primary outcome of 0.1 or less and provide the DSMC with little evidence of a benefit for any of the secondary parameters, the DSMC will consider stopping enrollment in the trial for futility. Details for these futility analyses will be included in the DSMC monitoring plan. Patients who have been enrolled in the study will be followed through the 22 - 26month evaluation.

(2) Slow patient enrollment: A timeline for enrollment is proposed, above, based on eligible patients anticipated at the NRN centers. This will be monitored, and if patient enrollment does not meet expected, the reasons for the problem will be analyzed and the potential for study completion assessed. Study forms will be constructed to include reasons for lack of enrollment, to assist in this process.

<u>BUDGET</u> (not including RTI costs) based on 800 patients

Pharmacy cost (avg):	$2000/center set-up fee + $500/year x 3 years =	$ 56,000
	Per patient (28 doses x $20/dose) =	$448,000*
Coordinator time:	10 hours/patient @ $35/hr ($350/pt)	$280,000
Follow up (est. deaths 15%, follow up rate 90% = 612):		
	$400 GDB babies, $700/pt otherwise (est.2/3)	<u>$367,200</u>
Estimated total:		$1,151,200

(*This may be lower, since all doses for each day can be prepared and dispensed at one time)

REFERENCES

1. Morley CJ, Davis PG, Doyle LW et al, COIN Trial Investigators. Nasal CPAP or intubation at birth for very preterm infants. N Engl J Med 2008; 358:700-8.
2. Walsh M, Laptook A, Kazzi SN, et al (National Institute of Child Health and Human Development Neonatal Research Network). A cluster-randomized trial of benchmarking and multimodal quality improvement to improve rates of survival free of bronchopulmonary dysplasia for infants with birth weights of less than 1250 grams. Pediatrics. 2007; 119:876-90.
3. Vohr BR, Wright LL, Poole WK, McDonald SA. Neurodevelopmental outcomes of extremely low birth weight infants <32 weeks' gestation between 1993 and 1998. Pediatrics. 2005; 116:635-43.
4. Walsh MC, Yao Q, Horbar JD, et al. Changes in the use of postnatal steroids for bronchopulmonary dysplasia in 3 large neonatal networks. Pediatrics 2006; 118:e1328-35.
5. Shinwell ES, Lerner-Geva L, Lusky A, Reichman B. Less postnatal steroids, more bronchopulmonary dysplasia: a population-based study in very low birthweight infants. Arch Dis Child Fetal Neonatal Ed. 2007; 92:F30-3.
6. Kobaly K, Schluchter M, Minich N, et al. Outcomes of extremely low birth weight (<1 kg) and extremely low gestational age (<28 weeks) infants with bronchopulmonary dysplasia: effects of practice changes in 2000 to 2003. Pediatrics. 2008; 121:73-81.
7. Yoder BA, Harrison M, Clark RH. Time-related changes in steroid use and bronchopulmonary dysplasia in preterm infants. Pediatrics. 2009; 124:673-9.
8. Halliday HL, Ehrenkranz RA, Doyle LW. Early (< 8 days) postnatal corticosteroids for preventing chronic lung disease in preterm infants. Cochrane Database Syst Rev. 2009 Jan 21; (1):CD001146
9. Halliday HL, Ehrenkranz RA, Doyle LW. Late (>7 days) postnatal corticosteroids for chronic lung disease in preterm infants. Cochrane Database Syst Rev. 2009 Jan 21; (1):CD001145.
10. Barrington KJ. The adverse neuro-developmental effects of postnatal steroids in the preterm infant: a systematic review of RCTs. BMC Pediatr 2001; 1:1.
11. Yeh TF, Lin YJ, Lin HC, et al. Outcomes at school age after postnatal dexamethasone therapy for lung disease of prematurity. N Engl J Med 2004; 350:1304-13.
12. American Academy of Pediatrics Committee on Fetus and Newborn, Canadian Paediatric Society Fetus and Newborn Committee. Postnatal corticosteroids to treat or prevent chronic lung disease in preterm infants. Pediatrics 2002; 109:330-8.
13. Doyle LW, Halliday HL, Ehrenkranz RA, et al. Impact of postnatal systemic corticosteroids on mortality and cerebral palsy in preterm infants: effect modification by risk for chronic lung disease. Pediatrics. 2005; 115:655-61.
14. McEvoy C, Bowling S, Williamson K, McGaw P, Durand M. Randomized, double-blinded trial of low-dose dexamethasone: II. Functional residual capacity and pulmonary outcome in very low birth weight infants at risk for bronchopulmonary dysplasia. Pediatr Pulmonol. 200; 38:55-63.

15. Doyle LW, Davis PG, Morley CJ, et al. (DART Study Investigators). Low-dose dexamethasone facilitates extubation among chronically ventilator-dependent infants: a multicenter, international, randomized, controlled trial. Pediatrics. 2006; 117:75-83.
16. Doyle LW, Davis PG, Morley CJ, et al (DART Study Investigators). Outcome at 2 years of age of infants from the DART study: a multicenter, international, randomized, controlled trial of low-dose dexamethasone. Pediatrics. 2007; 119:716-21.
17. Stark AR, Carlo W, Vohr BR, et al, *NICHD Neonatal Research Network.* Neurodevelopmental outcome and growth at 18 – 22 months. Pediatr Res 2001; 49:388A (abstract).
18. van der Heide-Jalving M, Kamphuis PJ, van der Laan MJ, et al. Short- and long-term effects of neonatal glucocorticoid therapy: is hydrocortisone an alternative to dexamethasone? Acta Paediatr 2003; 92:827-35.
19. Peltoniemi OM, Lano A, Puosi R, Yliherva A, Bonsante F, Kari MA, Hallman M. Trial of early neonatal hydrocortisone: two-year follow-up. Neonatology. 2008; 95:240-247.
20. Watterberg KL, Shaffer ML, Mishefske MJ, et al. Growth and neurodevelopmental outcomes after early low-dose hydrocortisone treatment in extremely low birth weight infants. Pediatrics 2007; 120: 40 - 48.
21. Rademaker KJ, Uiterwaal CS, Groenendaal F, et al. Neonatal hydrocortisone treatment: neurodevelopmental outcome and MRI at school age in preterm-born children. J Pediatr. 2007; 150:351-7.
22. Parikh NA, Lasky R, Tyson J, Kennedy K, Zhang Y, Yu X. Does low dose hydrocortisone improve volumetric MRI measures of cerebral growth in extremely low birth weight infants at high risk for bronchopulmonary dysplasia?: a randomized trial. E-PAS2009:3212.2
23. McEwen BS. The brain is an important target of adrenal steroid actions: a comparison of synthetic and natural steroids. Ann NY Acad Sci 1997; 823: 201-13.
24. De Kloet RE, Vreugdenhil E, Oitzl MS, Joels M. Brain corticosteroid receptor balance in health and disease. Endocrine Rev 1998; 19:269-301.
25. Sloviter RS, Sollas AL, Neubort S. Hippocampal dentate granule cell degeneration after adrenalectomy in the rat is not reversed by dexamethasone. Brain Res 1995; 682: 227-30.
26. Hassan AHS, von Rosenstiel P, Patchev VK, et al. Exacerbation of apoptosis in the dentate gyrus of the aged rat by dexamethasone and the protective role of corticosterone. Exp Neurol 1996; 140:43-52.
27. Isaacs EB, Lucas A, Chong WK, et al. Hippocampal volume and everyday memory in children of very low birth weight. Pediatr Res 2000; 47: 713-720.
28. Giménez M, Junqué C, Narberhaus A, et al. Hippocampal gray matter reduction associates with memory deficits in adolescents with history of prematurity. Neuroimage 2004; 23:869-77.
29. Nosarti C, Al-Asady MH, Frangou S, Stewart AL, Rifkin L, Murray RM. Adolescents who were born very preterm have decreased brain volumes. Brain. 2002; 125:1616-23.
30. Murphy BP, Inder TE, Huppi PS et al. Impaired cerebral cortical gray matter growth after treatment with dexamethasone for neonatal chronic lung disease. Pediatrics 2001; 107: 217-21.
31. Parikh NA, Lasky RE, Kennedy KA, et al. Postnatal dexamethasone therapy and cerebral tissue volumes in extremely low birth weight infants. Pediatrics. 2007; 119:265-72.

32. Thompson DK, Wood SJ, Doyle LW, et al. Neonate hippocampal volumes: prematurity, perinatal predictors, and 2-year outcome. Ann Neurol 2008; 63:642-51.
33. Rademaker KJ, Rijpert M, Uiterwaal CS, et al. Neonatal hydrocortisone treatment related to 1H-MRS of the hippocampus and short-term memory at school age in preterm born children. Pediatr Res. 2006; 59:309-13.
34. Attridge JT, Clark R, Walker MW, Gordon PV. New insights into spontaneous intestinal perforation using a national data set: (2) two populations of patients with perforations. J Perinatol 2006; 26:185-8.
35. Finer NN, Powers RJ, Ou CH, Durand D, Wirtschafter D, Gould JB; California Perinatal Quality Care Collaborative Executive Committee. Prospective evaluation of postnatal steroid administration: a 1-year experience from the California Perinatal Quality Care Collaborative. Pediatrics. 2006 Mar;117(3):704-13.
36. Yeager MP, Rassias AJ, Fillinger MP, Discipio AW, Gloor KE, Gregory JA, Guyre PM. Cortisol antiinflammatory effects are maximal at postoperative plasma concentrations. Crit Care Med. 2005; 33:1507-12.
37. Pejovic B, Peco-Antic A, Marinkovic-Eric J. Blood pressure in non-critically ill preterm and full-term neonates. Pediatr Nephrol. 2007; 22:249-57.
38. Watterberg KL, Gerdes JS, Cole CH et al. Prophylaxis of early adrenal insufficiency to prevent bronchopulmonary dysplasia: a multicenter trial. Pediatrics 2004; 114:1649-57.
39. Peltoniemi O, Kari MA, Heinonen K et al. Pretreatment cortisol values may predict responses to hydrocortisone administration for the prevention of bronchopulmonary dysplasia in high-risk infants. J Pediatr 2005; 146: 632-7.
40. Bonsante F, Latorre G, Iacobelli S, Forziati V, Laforgia N, Esposito L, Mautone A. Early low-dose hydrocortisone in very preterm infants: a randomized, placebo-controlled trial. Neonatology. 2007;91:217-21.

Summary of Hydrocortisone for BPD Protocol Revisions. Note: Only those changes that required revisions to the Protocol are listed below. Other technical clarifications and/or notifications that did not require revisions are omitted.

Date/Version	Affected Section(s)	Summary of Revisions Made	Rationale	Technical Memo
05/7/12 (v1 to v2)	Sections noting the follow-up window and section 8: Analysis of study outcomes, (a) Study specifications and definitions	1. The study follow-up window was updated to 22-26 months corrected age.<br>2. The NDI definition was revised as follows:<br>(v) "Severe neurodevelopmental impairment" will be defined as (1) a BSID-III cognitive score or BSID-III motor score more than 2 SD below the mean (<70); OR (2) a Gross Motor Function classification code of ~~3~~ 4 – 5 (Level 2 or 3) indicating moderate to severe cerebral palsy; OR (3) severe to profound hearing impairment ~~requiring hearing aids~~ (no functional hearing with amplification); OR (4) bilateral blindness that cannot be corrected.<br>(vi) "Moderate neurodevelopmental impairment" will be defined as (1) BSID-III cognitive score or BSID-III motor score 1 – 2 SD below the mean (70 – 84); OR (2) a GMFCS code of 2 -3 (Possible Level 1 or Level 1) indicating no cerebral palsy or mild cerebral palsy; OR (3) ~~a~~ moderate hearing impairment (can hear and follow directions with amplification); OR (4) moderate vision impairment (limited vision with correction or unilateral blindness).	The Neonatal Research Network (NRN) Steering Committee voted to update the follow-up window from 18-22 months to 22-26 months corrected age. Additionally, the NDI definition was updated.	#7
08/27/13 (v2 to v3)	Methods and Procedures<br><br>8 Analysis of study outcomes, (a) Study specifications and definitions	Hydrocortisone treatment for hypotension was clarified to exclude infants receiving not only hydrocortisone but other systemic glucocorticoids for more than 14 days or within 7 days of study entry.<br><br>The BITSEA was removed from the follow-up assessments	The Neonatal Research Network (NRN) voted to replace the BITSEA with the CBCL for all trial and 2 year follow-up.	#10
