# Supplementary material for: Heterogeneity of Treatment Effects of Hydrocortisone by Risk of Bronchopulmonary Dysplasia or Death Among Extremely Preterm Infants in the National Institute of Child Health and Human Development Neonatal Research Network Trial: A Secondary Analysis of a Randomized Clinical Trial
Source: JAMA Netw Open. 2023 May 31;6(5):e2315315. doi: 10.1001/jamanetworkopen.2023.15315 (PMC10233424; doi:10.1001/jamanetworkopen.2023.15315)
Supplement: Supplement 2. — eTable 1. Demographic and Clinical Characteristics Used for Risk Estimates by Quartile of Baseline Risk for Grades 2 to 3 BPD or Death and Exposure Group eTable 2. Additional Clinical Characteristics by Quartile of Baseline Risk for Grades 2 to 3 BPD or Death eTable 3. Additional Clinical Characteristics by Quartile of Baseline Risk for Grades 2 to 3 BPD or Death and Exposure Group eFigure 1. CONSORT Flow Diagram eFigure 2. Distribution of Predicted Probability of Grades 2 to 3 BPD or Death in the Enrolled Population eFigure 3. Calibration Plot of Association of Estimated Probability of Grades 2 to 3 BPD or Death to the Observed Outcome of Grades 2 to 3 BPD or Death [file jamanetwopen-e2315315-s002.pdf]

Supplementary Online Content

Gentle JS, Rysavy MA, Li L, et al; National Institute of Child Health and Human Development Neonatal Research Network. Heterogeneity of treatment effects of hydrocortisone by risk of bronchopulmonary dysplasia or death among extremely preterm infants in the National Institute of Child Health and Human Development Neonatal Research Network Trial: a secondary analysis of a randomized clinical trial. *JAMA Netw Open*. 2023;6(5):e2315315. doi:10.1001/jamanetworkopen.2023.15315

eTable 1. Demographic and Clinical Characteristics Used for Risk Estimates by Quartile of Baseline Risk for Grades 2 to 3 BPD or Death and Exposure Group
eTable 2. Additional Clinical Characteristics by Quartile of Baseline Risk for Grades 2 to 3 BPD or Death
eTable 3. Additional Clinical Characteristics by Quartile of Baseline Risk for Grades 2 to 3 BPD or Death and Exposure Group
eFigure 1. CONSORT Flow Diagram
eFigure 2. Distribution of Predicted Probability of Grades 2 to 3 BPD or Death in the Enrolled Population
eFigure 3. Calibration Plot of Association of Estimated Probability of Grades 2 to 3 BPD or Death to the Observed Outcome of Grades 2 to 3 BPD or Death

This supplementary material has been provided by the authors to give readers additional information about their work.

© 2023 Gentle SJ et al. *JAMA Network Open*.

eTable 1. Demographic and Clinical Characteristics Used for Risk Estimates by Quartile of Baseline Risk for Grades 2 to 3 BPD or Death and Exposure Group

	Quartile 1<br>18-45%		Quartile 2<br>46-53%		Quartile 3<br>54-65%		Quartile 4<br>66-84%		All	
	HC	Placebo	HC	Placebo	HC	Placebo	HC	Placebo	HC	Placebo
	n=110	n=89	n=89	n=111	n=99	n=101	n=100	n=100	n=398	n=401
Gestational age (wks) median (IQR)	25<br>(24-26)	25<br>(24-26)	25<br>(24-25)	25<br>(24-26)	24<br>(24-26)	25<br>(24-26)	25<br>(24-26)	25<br>(24-26)	25<br>(24-26)	25<br>(24-26)
Birth weight (g) median (IQR)	773<br>(660-860)	848<br>(720-984)	700<br>(611-789)	730<br>(621-790)	665<br>(580-770)	670<br>(585-770)	633<br>(540-710)	630<br>(520-710)	690 (600-799)	695 (600-810)
Male: n/N (%)	26/110 (24)	32/89<br>(36)	47/89<br>(53)	72/111<br>(65)	55/99<br>(56)	66/101<br>(65)	58/100<br>(58)	65/100<br>(65)	186/398<br>(47)	235/401<br>(59)
Highest FiO_2 on Day 14: median (IQR)	36<br>(30-44)	35<br>(30-41)	46<br>(38-55)	38<br>(32-50)	48<br>(38-62)	48<br>(36-60)	60<br>(50-80)	60<br>(45-76)	46<br>(35-62)	44<br>(34-60)
Respiratory support on postnatal day 14:										
Conventional Ventilation	2 (2)	2 (2)	7 (8)	6 (5)	39 (39)	29 (29)	83 (83)	82 (82)	131 (33)	119 (30)
High frequency ventilation	106 (96)	87 (98)	82 (92)	105 (95)	60 (61)	72 (71)	17 (17)	18 (18)	265 (67)	282 (70)
Noninvasive positive pressure ventilation	2 (2)	2 (2)	2 (2)	2 (2)	2 (2)	2 (2)	2 (2)	2 (1.8)	2 (2)	2 (2)
Surgical NEC (%)	0 (0)	1 (1)	2 (2)	5 (5)	6 (6)	9 (9)	7 (7)	10 (10)	15 (4)	25 (6)

BPD: bronchopulmonary dysplasia; FiO_2: fraction of inspired oxygen; NEC: necrotizing enterocolitis

© 2023 Gentle SJ et al. *JAMA Network Open*.

eTable 2. Additional Clinical Characteristics by Quartile of Baseline Risk for Grades 2 to 3 BPD or Death

	Quartile 1 18-45%	Quartile 2 46-53%	Quartile 3 54-65%	Quartile 4 66-84%	All
	n=199	n=200	n=200	n=200	n=799
Days of mechanical ventilation[a]: median (IQR)	30 (21-43)	37 (29-51)	42 (30-57)	46 (35-65)	38 (28-54)
Highest FiO_2 at enrollment: median (IQR)	33 (30-40)	40 (34-48)	50 (40-61)	70 (60-97)	46 (35-61)
Extubation failure[a]: n/N (%)	16 (8)	28 (14)	27 (15)	38 (21)	109 (15)
Open label dexamethasone exposure: n/N (%)	56 (29)	64 (33)	92 (51)	94 (53)	306 (41)
Clinical chorioamnionitis: n/N (%)	36 (18)	43 (22)	29 (15)	22 (11)	130 (16)
Surgical PDA treatment: n/N (%)	30 (15)	40 (20)	27 (14)	27 (14)	124 (16)
Pharmacological PDA treatment: n/N (%)	96 (48)	91 (46)	87 (44)	82 (41)	356 (45)
Early onset sepsis: n/N (%)	2 (1)	2 (1)	7 (4)	3 (2)	14 (2)
Late onset sepsis: n/N (%)	37 (19)	63 (32)	68 (34)	70 (35)	238 (30)

BPD: bronchopulmonary dysplasia; FiO_2: fraction of inspired oxygen; PDA: patent ductus arteriosus

© 2023 Gentle SJ et al. *JAMA Network Open*.

eTable 3. Additional Clinical Characteristics by Quartile of Baseline Risk for Grades 2 to 3 BPD or Death and Exposure Group

	Quartile 1 18-45%		Quartile 2 46-53%		Quartile 3 54-65%		Quartile 4 66-84%		All	
	HC	Placebo	HC	Placebo	HC	Placebo	HC	Placebo	HC	Placebo
	n=110	n=89	n=89	n=111	n=99	n=101	n=100	n=100	n=398	n=401
Days of mechanical ventilation[a]: median (IQR)	33 (23-46)	29 (20-40)	36 (27-51)	37 (30-51)	40 (27-56)	44.5 (33-58)	43 (31-60)	50 (38-66)	37 (27-54)	40 (30-55)
Highest FiO_2 at enrollment: median (IQR)	32.5 (30-40)	33 (30-39)	45 (35-50)	39 (34-46)	51 (41-62)	50 (40-60)	73 (59-100)	70 (60-87)	47 (35-63)	45 (35-60)
Extubation failure[a]: n/N (%)	11/109 (10)	5/87 (6)	13/88 (15)	15/108 (14)	13/90 (14)	14/89 (16)	21/91 (23)	17/88 (19)	58/378 (15)	51/372 (14)
Open label dexamethasone exposure: n/N (%)	35/109 (32)	21/87 (24)	27/88 (31)	37/108 (34)	42/90 (47)	50/89 (56)	46/91 (51)	48/88 (55)	150/378 (40)	156/372 (42)
Clinical chorioamnionitis: n/N (%)	24/109 (22)	12/89 (14)	19/89 (21)	24/111 (22)	12/99 (12)	17/100 (17)	10/100 (10)	12/100 (12)	65/397 (16)	65/400 (16)
Surgical PDA treatment: n/N (%)	21/110 (19)	9/89 (10)	22/89 (25)	18/111 (16)	11/99 (11)	16/101 (16)	13/100 (13)	14/100 (14)	67/398 (17)	57/401 (14)
Pharmacological PDA treatment: n/N (%)	58/110 (53)	38/89 (43)	39/89 (44)	52/111 (47)	39/99 (39)	48/101 (48)	37/100 (37)	45/100 (45)	173/398 (44)	183/401 (46)
Early onset sepsis: n/N (%)	2/110 (2)	0/89 (0)	1/89 (1)	1/111 (1)	5/99 (5)	2/101 (2)	1/100 (1)	2/100 (2)	9/398 (2)	5/401(1)
Late onset sepsis: n/N (%)	16/110 (15)	21/89 (24)	30/89 (34)	33/111 (28)	37/99 (37)	31/101 (31)	38/100 (38)	32/100 (32)	121/398 (30)	117/401 (29)

[a]Among surviving infants by 36 weeks' postmenstrual age; PDA: patent ductus arteriosus

© 2023 Gentle SJ et al. *JAMA Network Open*.

eFigure 1. CONSORT Flow Diagram

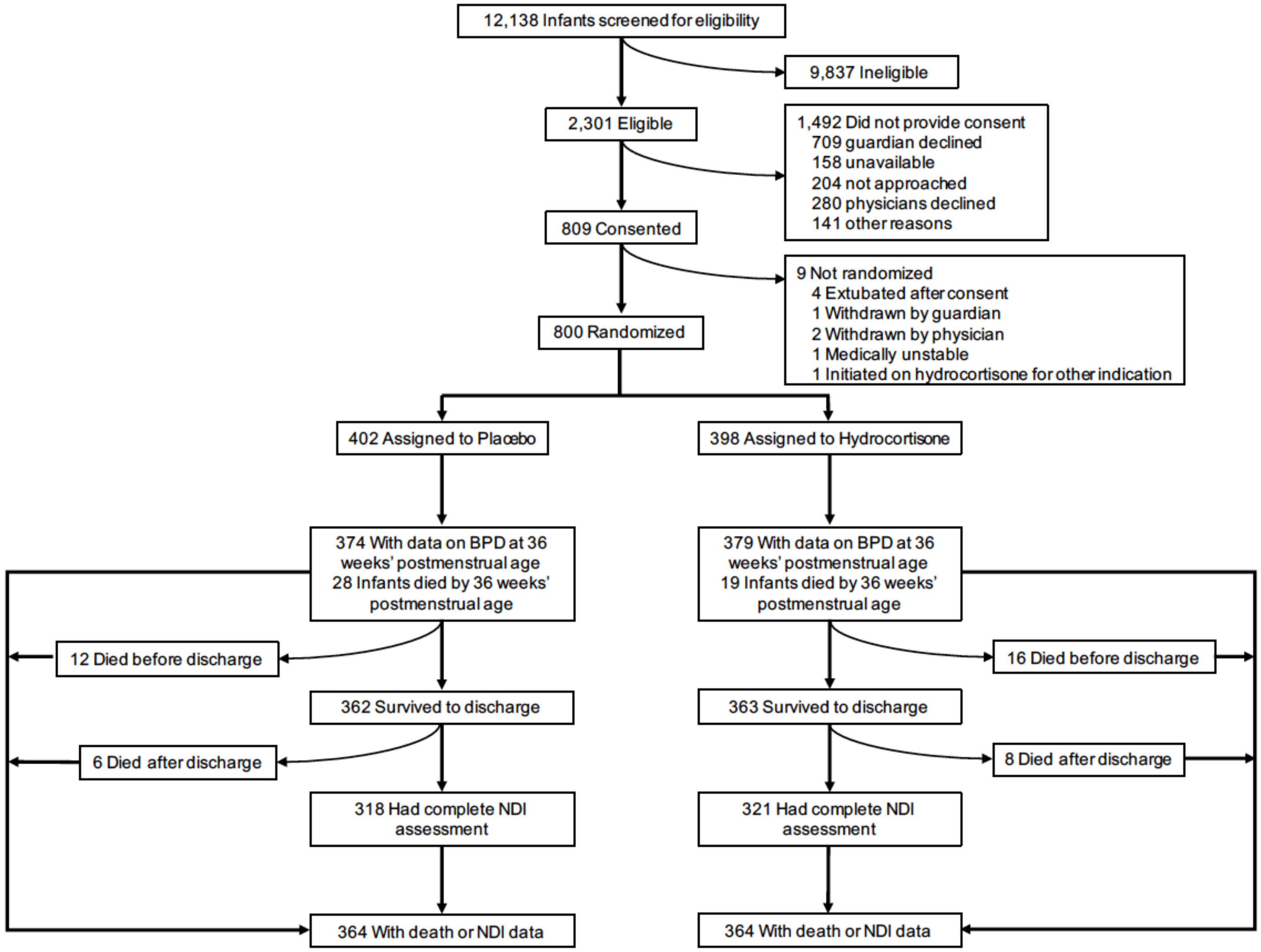

eFigure1. CONSORT Flow Diagram detailing eligible, consented, and randomized infants followed by infants available for analysis of both safety and efficacy outcomes.

© 2023 Gentle SJ et al. *JAMA Network Open*.

eFigure 2. Distribution of Predicted Probability of Grades 2 to 3 BPD or Death in the Enrolled Population

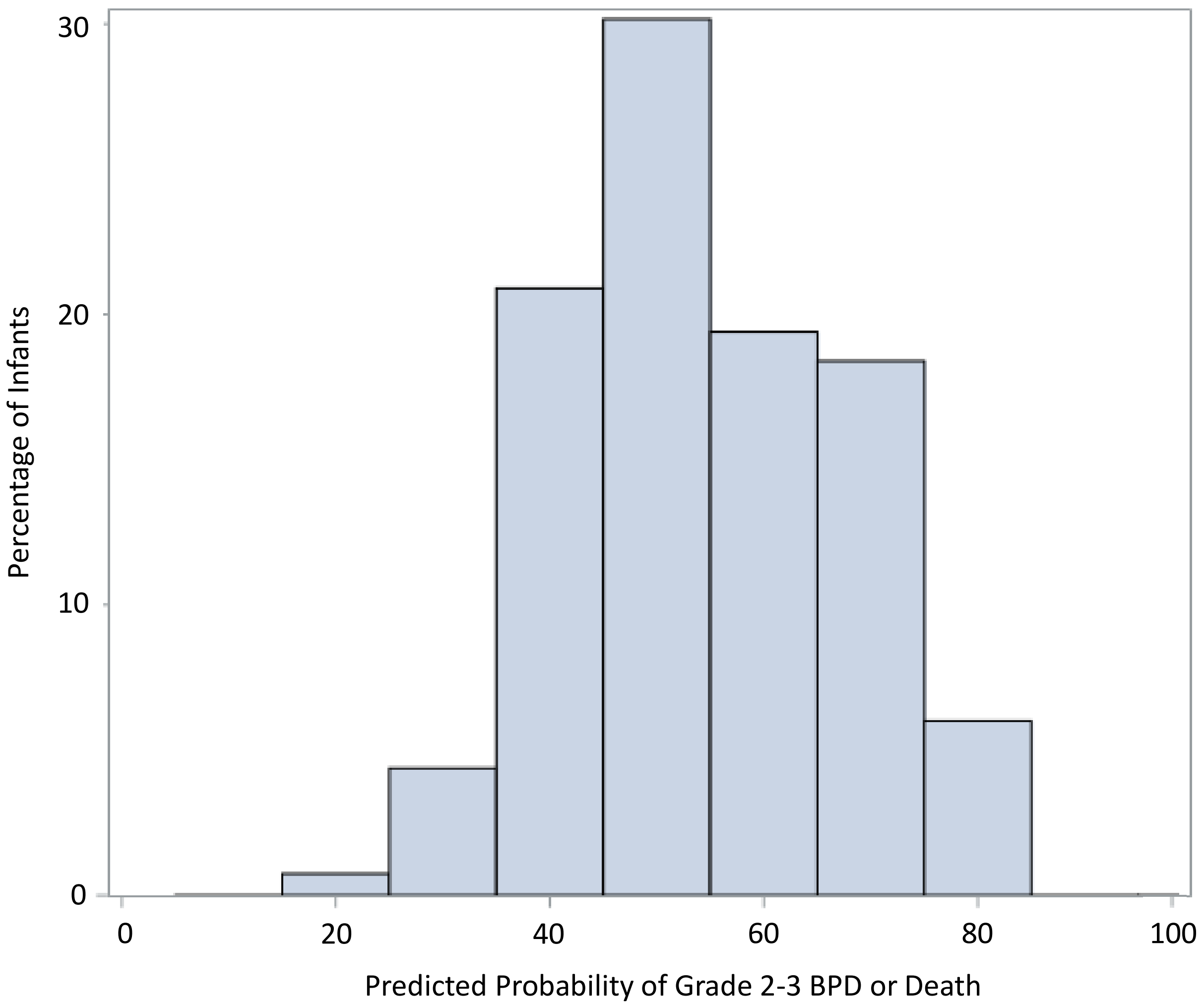

eFigure 2. Distribution of predicted probability of grade 2-3 BPD or death in the enrolled population. The mean predicted probability was 55% (range 18-84%).

© 2023 Gentle SJ et al. *JAMA Network Open*.

eFigure 3. Calibration Plot of Association of Estimated Probability of Grades 2 to 3 BPD or Death to the Observed Outcome of Grades 2 to 3 BPD or Death

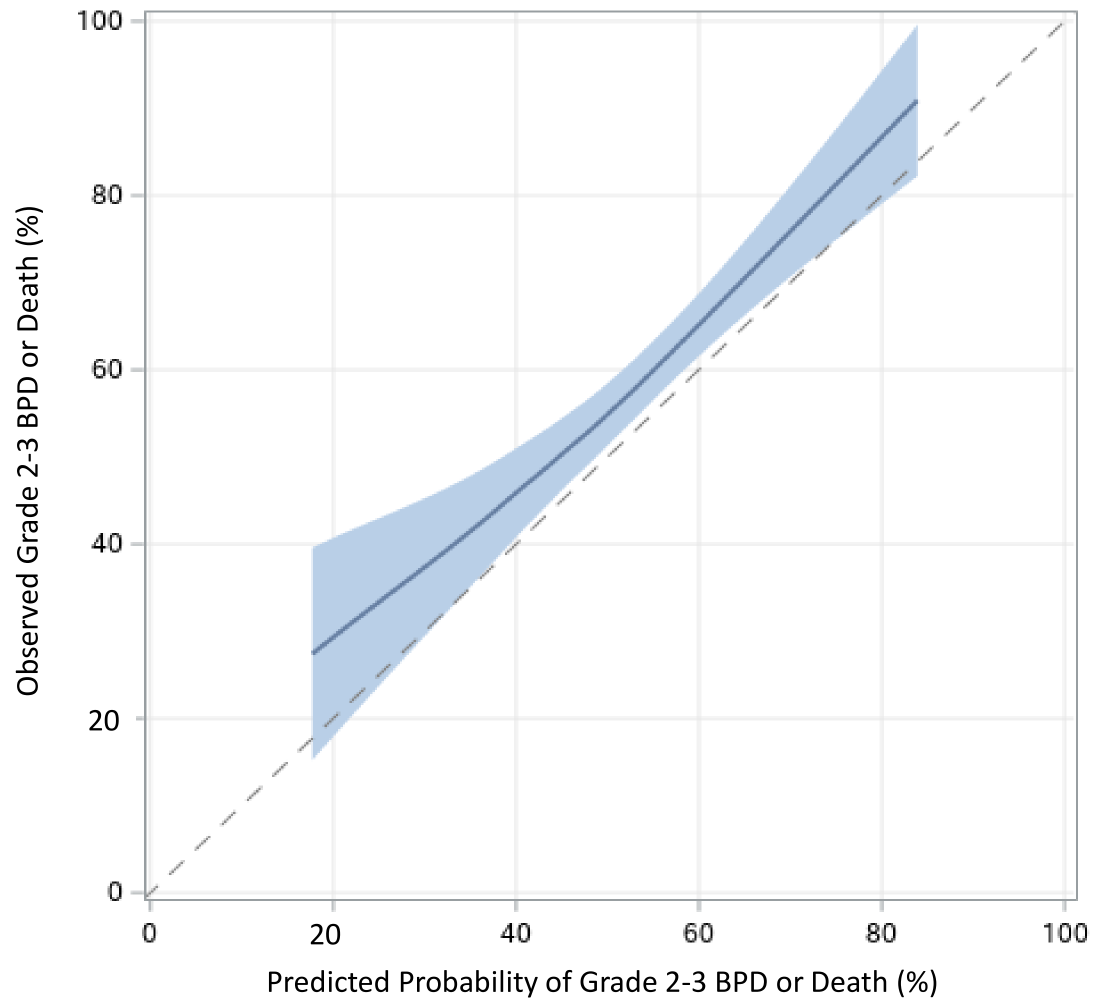

eFigure 3. Calibration plot relating predicted probability of grade 2-3 BPD or death to the observed outcome of grade 2-3 BPD or death. The ratio of observed to expected outcomes was 1.09.

© 2023 Gentle SJ et al. *JAMA Network Open*.
