# Supplementary material for: Heterogeneity of Treatment Effects of Hydrocortisone by Risk of Bronchopulmonary Dysplasia or Death Among Extremely Preterm Infants in the National Institute of Child Health and Human Development Neonatal Research Network Trial: A Secondary Analysis of a Randomized Clinical Trial
Source: JAMA Netw Open. 2023 May 31;6(5):e2315315. doi: 10.1001/jamanetworkopen.2023.15315 (PMC10233424; doi:10.1001/jamanetworkopen.2023.15315)
Supplement: Supplement 3. — Nonauthor Collaborators [file jamanetwopen-e2315315-s003.pdf]

Supplemental Online Content: Nonauthor Collaborators
*First name, last name, and suffix (if applicable) are required and will appear in PubMed.

*Group Name(s): National Institute of Child Health and Human Development Neonatal Research Network								
***First Name and Middle Initial(s)**	***Last Name**	***Suffix (eg, Jr, III)**	Academic Degrees	Institution	Location (city, state/province, country)	Role or Contribution, eg, chair, principal investigator	Group (if more than 1 Group listed in the byline) and/or Subgroup (eg, Steering Committee)	
Abbot R	Laptook		MD	Alpert Medical School of Brown University and Women & Infants Hospital of Rhode Island	Providence, RI	investigator		
Martin	Keszler		MD	Alpert Medical School of Brown University and Women & Infants Hospital of Rhode Island	Providence, RI	investigator		
Betty R	Vohr		MD	Alpert Medical School of Brown University and Women & Infants Hospital of Rhode Island	Providence, RI	investigator		
Angelita M	Hensman		PhD RNC-NIC	Alpert Medical School of Brown University and Women & Infants Hospital of Rhode Island	Providence, RI	assisted with patient enrollment and data collection		
Elisa	Vieira		BSN RN	Alpert Medical School of Brown University and Women & Infants Hospital of Rhode Island	Providence, RI	assisted with patient enrollment and data collection		
Lucille	St. Pierre		BSN RN	Alpert Medical School of Brown University and Women & Infants Hospital of Rhode Island	Providence, RI	assisted with patient enrollment and data collection		
Robert T	Burke		MD, MPH	Alpert Medical School of Brown University and Women & Infants Hospital of Rhode Island	Providence, RI	assisted with patient enrollment and data collection		
Barbara	Alksinis		RNC PNP	Alpert Medical School of Brown University and Women & Infants Hospital of Rhode Island	Providence, RI	assisted with patient enrollment and data collection		
Melinda	Caskey		MD	Alpert Medical School of Brown University and Women & Infants Hospital of Rhode Island	Providence, RI	investigator		
Laurie	Hoffman		MD	Alpert Medical School of Brown University and Women & Infants Hospital of Rhode Island	Providence, RI	investigator		

*First name, last name, and suffix (if applicable) are required and will appear in PubMed.

***First Name and Middle Initial(s)**	***Last Name**	***Suffix (eg, Jr, III)**	Academic Degrees	Institution	Location (city, state/province, country)	Role or Contribution, eg, chair, principal investigator	Group (if more than 1 Group listed in the byline) and/or Subgroup (eg, Steering Committee)	
Katharine	Johnson		MD	Alpert Medical School of Brown University and Women & Infants Hospital of Rhode Island	Providence, RI	investigator		
Mary L	Keszler		MD	Alpert Medical School of Brown University and Women & Infants Hospital of Rhode Island	Providence, RI	investigator		
Andrea	Knoll			Alpert Medical School of Brown University and Women & Infants Hospital of Rhode Island	Providence, RI	assisted with patient enrollment and data collection		
Teresa M	Leach		Med CAES	Alpert Medical School of Brown University and Women & Infants Hospital of Rhode Island	Providence, RI	assisted with patient enrollment and data collection		
Emily	Little		BSN RN	Alpert Medical School of Brown University and Women & Infants Hospital of Rhode Island	Providence, RI	assisted with patient enrollment and data collection		
Elisabeth C	McGowan		MD	Alpert Medical School of Brown University and Women & Infants Hospital of Rhode Island	Providence, RI	investigator		
Bonnie E	Stephens		MD	Alpert Medical School of Brown University and Women & Infants Hospital of Rhode Island	Providence, RI	investigator		
Victoria E	Watson		MS CAS	Alpert Medical School of Brown University and Women & Infants Hospital of Rhode Island	Providence, RI	investigator		
Anna Maria	Hibbs		MD, MSCE	Case Western Reserve University, Rainbow Babies & Children's Hospital	Cleveland, OH	investigator		
Michele C	Walsh		MD, MS	Case Western Reserve University, Rainbow Babies & Children's Hospital	Cleveland, OH	investigator		
Deanne E	Wilson-Costello		MD	Case Western Reserve University, Rainbow Babies & Children's Hospital	Cleveland, OH	investigator		

Supplemental Online Content: Nonauthor Collaborators
*First name, last name, and suffix (if applicable) are required and will appear in PubMed.

***First Name and Middle Initial(s)**	***Last Name**	***Suffix (eg, Jr, III)**	Academic Degrees	Institution	Location (city, state/province, country)	Role or Contribution, eg, chair, principal investigator	Group (if more than 1 Group listed in the byline) and/or Subgroup (eg, Steering Committee)	
Nancy S	Newman		RN	Case Western Reserve University, Rainbow Babies & Children's Hospital	Cleveland, OH	assisted with patient enrollment and data collection		
Monika	Bhola		MD	Case Western Reserve University, Rainbow Babies & Children's Hospital	Cleveland, OH	investigator		
Allison H	Payne		MD, MS	Case Western Reserve University, Rainbow Babies & Children's Hospital	Cleveland, OH	investigator		
Bonnie S	Siner		RN	Case Western Reserve University, Rainbow Babies & Children's Hospital	Cleveland, OH	assisted with patient enrollment and data collection		
Gulgun	Yalcinkaya		MD	Case Western Reserve University, Rainbow Babies & Children's Hospital	Cleveland, OH	investigator		
William E	Truog		MD	Children's Mercy Hospital	Kansas City, MO	investigator		
Howard W	Kilbride		MD	Children's Mercy Hospital	Kansas City, MO	investigator		
Cheri	Gauldin		RN, BS CCRC	Children's Mercy Hospital	Kansas City, MO	assisted with patient enrollment and data collection		
Eugenia K	Pallotto		MD, MSCE	Children's Mercy Hospital	Kansas City, MO	investigator		
Anne	Holmes		RN, MSN, MBA-HCM CCRC	Children's Mercy Hospital	Kansas City, MO	assisted with patient enrollment and data collection		
Kathy	Johnson		RN, CCRC	Children's Mercy Hospital	Kansas City, MO	assisted with patient enrollment and data collection		
Allison	Scott		RNC-NIC BSN CCRC	Children's Mercy Hospital	Kansas City, MO	assisted with patient enrollment and data collection		
Brenda B	Poindexter		MD, MS	Cincinnati Children's Hospital Medical Center, University Hospital, and Good Samaritan Hospital	Cincinnati, OH	investigator		

*First name, last name, and suffix (if applicable) are required and will appear in PubMed.

***First Name and Middle Initial(s)**	***Last Name**	***Suffix (eg, Jr, III)**	Academic Degrees	Institution	Location (city, state/province, country)	Role or Contribution, eg, chair, principal investigator	Group (if more than 1 Group listed in the byline) and/or Subgroup (eg, Steering Committee)	
Kurt	Schibler		MD	Cincinnati Children's Hospital Medical Center, University Hospital, and Good Samaritan Hospital	Cincinnati, OH	investigator		
Stephanie	Merhar		MD, MS	Cincinnati Children's Hospital Medical Center, University Hospital, and Good Samaritan Hospital	Cincinnati, OH	investigator		
Kimberly	Yolton		PhD	Cincinnati Children's Hospital Medical Center, University Hospital, and Good Samaritan Hospital	Cincinnati, OH	assisted with patient enrollment and data collection		
Cathy	Grisby		BSN CCRC	Cincinnati Children's Hospital Medical Center, University Hospital, and Good Samaritan Hospital	Cincinnati, OH	assisted with patient enrollment and data collection		
Traci	Beiersdorfer		RN bsn	Cincinnati Children's Hospital Medical Center, University Hospital, and Good Samaritan Hospital	Cincinnati, OH	assisted with patient enrollment and data collection		
Tanya E	Cahill		MD	Cincinnati Children's Hospital Medical Center, University Hospital, and Good Samaritan Hospital	Cincinnati, OH	investigator		
Juanita	Dudley		RN BSN	Cincinnati Children's Hospital Medical Center, University Hospital, and Good Samaritan Hospital	Cincinnati, OH	assisted with patient enrollment and data collection		
Teresa L	Gratton		PA	Cincinnati Children's Hospital Medical Center, University Hospital, and Good Samaritan Hospital	Cincinnati, OH	assisted with patient enrollment and data collection		

*First Name and Middle Initial(s)	*Last Name	*Suffix (eg, Jr, III)	Academic Degrees	Institution	Location (city, state/province, country)	Role or Contribution, eg, chair, principal investigator	Group (if more than 1 Group listed in the byline) and/or Subgroup (eg, Steering Committee)	
Kristin	Kirker		CRC	Cincinnati Children's Hospital Medical Center, University Hospital, and Good Samaritan Hospital	Cincinnati, OH	assisted with patient enrollment and data collection		
Julia	Thompson		RN BSN	Cincinnati Children's Hospital Medical Center, University Hospital, and Good Samaritan Hospital	Cincinnati, OH	assisted with patient enrollment and data collection		
Sandra	Wuertz		RN BSN CLC	Cincinnati Children's Hospital Medical Center, University Hospital, and Good Samaritan Hospital	Cincinnati, OH	assisted with patient enrollment and data collection		
Richard A	Polin		MD	Columbia University	New York, NY	NRN Steering Committee Chair		
Michael	Cotton		MD MHS	Duke University School of Medicine	Durham, NC	investigator		
Ronald N	Goldberg		MD	Duke University School of Medicine	Durham, NC	investigator		
William F	Malcolm		MD	Duke University School of Medicine	Durham, NC	investigator		
Ricki F	Goldstein		MD	Duke University School of Medicine	Durham, NC	investigator		
Joanne	Finkle		RN JD	Duke University School of Medicine	Durham, NC	assisted with patient enrollment and data collection		
Patricia L	Ashley		MD	Duke University School of Medicine	Durham, NC	investigator		
Kimberley A	Fisher		PhD FNP-BC IBCLC	Duke University School of Medicine	Durham, NC	assisted with patient enrollment and data collection		
Kathryn E	Gustafson		PhD	Duke University School of Medicine	Durham, NC	assisted with patient enrollment and data collection		
Deesha	Mago-Shah		MD	Duke University School of Medicine	Durham, NC	investigator		
Mollie	Warren		MD	Duke University School of Medicine	Durham, NC	investigator		
Carl L	Bose		MD	Duke University School of Medicine	Durham, NC	investigator		
Janice	Bernhardt		MS RN	Duke University School of Medicine	Durham, NC	assisted with patient enrollment and data collection		
Gennie	Bose		RN	Duke University School of Medicine	Durham, NC	assisted with patient enrollment and data collection		
Janice	Wereszczak		CPNP-AC/PC	Duke University School of Medicine	Durham, NC	assisted with patient enrollment and data collection		
Diane	Warner		MD MPH	Duke University School of Medicine	Durham, NC	investigator		

Supplemental Online Content: Nonauthor Collaborators
*First name, last name, and suffix (if applicable) are required and will appear in PubMed.

***First Name and Middle Initial(s)**	***Last Name**	***Suffix (eg, Jr, III)**	Academic Degrees	Institution	Location (city, state/province, country)	Role or Contribution, eg, chair, principal investigator	Group (if more than 1 Group listed in the byline) and/or Subgroup (eg, Steering Committee)	
Cindy	Clark		RN	Duke University School of Medicine	Durham, NC	assisted with patient enrollment and data collection		
Stephen D	Kicklighter		MD	Duke University School of Medicine	Durham, NC	investigator		
Alexandra	Bentley		MD	Duke University School of Medicine	Durham, NC	investigator		
Laura	Edwards		MD	Duke University School of Medicine	Durham, NC	investigator		
Ginger	Rhodes-Ryan		ARNP MSN,	Duke University School of Medicine	Durham, NC	assisted with patient enrollment and data collection		
Donna	White		RN-BC BSN	Duke University School of Medicine	Durham, NC	assisted with patient enrollment and data collection		
David P	Carlton		MD	Emory University	Atlanta, GA	investigator		
Barbara J	Stoll		MD	Emory University	Atlanta, GA	investigator		
Ellen C	Hale		B RN CCRC	Emory University	Atlanta, GA	assisted with patient enrollment and data collection		
Yvonne	Loggins		RN	Emory University	Atlanta, GA	assisted with patient enrollment and data collection		
Diane	Bottcher		RN	Emory University	Atlanta, GA	assisted with patient enrollment and data collection		
Sheena L	Carter		PhD	Emory University	Atlanta, GA	assisted with patient enrollment and data collection		
Salathiel	Kendrick-Allywood		MD	Emory University	Atlanta, GA	investigator		
Maureen	Mulligan LaRossa		RN	Emory University	Atlanta, GA	assisted with patient enrollment and data collection		
Colleen	Mackie		RRT	Emory University	Atlanta, GA	assisted with patient enrollment and data collection		
Gloria	Smikle		PNP	Emory University	Atlanta, GA	assisted with patient enrollment and data collection		
Lynn C	Comerford		NNP	Emory University	Atlanta, GA	assisted with patient enrollment and data collection		
Judith	Laursen		RN	Emory University	Atlanta, GA	assisted with patient enrollment and data collection		
Amy	Sanders		PsyD	Emory University	Atlanta, GA	assisted with patient enrollment and data collection		
Brenda B	Poindexter		MD MS	Emory University	Atlanta, GA	investigator		
Nathalie L	Maitre		MD PhD	Emory University	Atlanta, GA	investigator		
Ira	Adams-Chapman		MD	Emory University	Atlanta, GA	investigator		
Andrew A	Bremer		MD PhD	*Eunice Kennedy Shriver* National Institute of Child Health and Human Development	Bethesda, MD	investigator		
Stephanie	Wilson Archer		MA	*Eunice Kennedy Shriver* National Institute of Child Health and Human Development	Bethesda, MD	assisted with patient enrollment and data collection		

Supplemental Online Content: Nonauthor Collaborators
*First name, last name, and suffix (if applicable) are required and will appear in PubMed.

***First Name and Middle Initial(s)**	***Last Name**	***Suffix (eg, Jr, III)**	Academic Degrees	Institution	Location (city, state/province, country)	Role or Contribution, eg, chair, principal investigator	Group (if more than 1 Group listed in the byline) and/or Subgroup (eg, Steering Committee)	
Rosemary D	Higgins		MD	*Eunice Kennedy Shriver* National Institute of Child Health and Human Development	Bethesda, MD	investigator		
Jon E	Tyson		MD MPH	McGovern Medical School at The University of Texas Health Science Center at Houston	Houston, TX	investigator		
Amir M	Khan		MD	McGovern Medical School at The University of Texas Health Science Center at Houston	Houston, TX	investigator		
Kathleen A	Kennedy		MPD MPH	McGovern Medical School at The University of Texas Health Science Center at Houston	Houston, TX	investigator		
Barbara J	Stoll		MD	McGovern Medical School at The University of Texas Health Science Center at Houston	Houston, TX	investigator		
Ricardo A	Mosquera		MD MS	McGovern Medical School at The University of Texas Health Science Center at Houston	Houston, TX	investigator		
Andrea F	Duncan		MD	McGovern Medical School at The University of Texas Health Science Center at Houston	Houston, TX	investigator		
Georgia E	McDavid		RN	McGovern Medical School at The University of Texas Health Science Center at Houston	Houston, TX			
Nora	Alaniz		BS	McGovern Medical School at The University of Texas Health Science Center at Houston	Houston, TX	assisted with patient enrollment and data collection		
Elizabeth	Allain		Phd	McGovern Medical School at The University of Texas Health Science Center at Houston	Houston, TX	assisted with patient enrollment and data collection		
Julie	Arldt-McAlister		RN BSN	McGovern Medical School at The University of Texas Health Science Center at Houston	Houston, TX	assisted with patient enrollment and data collection		

*First name, last name, and suffix (if applicable) are required and will appear in PubMed.

***First Name and Middle Initial(s)**	***Last Name**	***Suffix (eg, Jr, III)**	Academic Degrees	Institution	Location (city, state/province, country)	Role or Contribution, eg, chair, principal investigator	Group (if more than 1 Group listed in the byline) and/or Subgroup (eg, Steering Committee)	
Debasree	Sana Boral		MD	McGovern Medical School at The University of Texas Health Science Center at Houston	Houston, TX	investigator		
Katrina	Burson		RN BSN	McGovern Medical School at The University of Texas Health Science Center at Houston	Houston, TX	assisted with patient enrollment and data collection		
Allison G	Dempsey		PhD	McGovern Medical School at The University of Texas Health Science Center at Houston	Houston, TX	assisted with patient enrollment and data collection		
Elizabeth	Eason		MD	McGovern Medical School at The University of Texas Health Science Center at Houston	Houston, TX	investigator		
Patricia W	Evans		MD	McGovern Medical School at The University of Texas Health Science Center at Houston	Houston, TX	investigator		
Carmen	Garcia		RN CCRP	McGovern Medical School at The University of Texas Health Science Center at Houston	Houston, TX	assisted with patient enrollment and data collection		
Charles	Green		PhD	McGovern Medical School at The University of Texas Health Science Center at Houston	Houston, TX	assisted with patient enrollment and data collection		
Donna J	Hall		RN	McGovern Medical School at The University of Texas Health Science Center at Houston	Houston, TX	assisted with patient enrollment and data collection		
Margarita	Jiminez		MD MPH	McGovern Medical School at The University of Texas Health Science Center at Houston	Houston, TX	investigator		
Janice	John		CPNP	McGovern Medical School at The University of Texas Health Science Center at Houston	Houston, TX	assisted with patient enrollment and data collection		
Patrick M	Jones		MD MA	McGovern Medical School at The University of Texas Health Science Center at Houston	Houston, TX	investigator		

*First name, last name, and suffix (if applicable) are required and will appear in PubMed.

***First Name and Middle Initial(s)**	***Last Name**	***Suffix (eg, Jr, III)**	Academic Degrees	Institution	Location (city, state/province, country)	Role or Contribution, eg, chair, principal investigator	Group (if more than 1 Group listed in the byline) and/or Subgroup (eg, Steering Committee)	
M. Layne	Lillie		RN BSN	McGovern Medical School at The University of Texas Health Science Center at Houston	Houston, TX	assisted with patient enrollment and data collection		
Karen	Martin		RN	McGovern Medical School at The University of Texas Health Science Center at Houston	Houston, TX	assisted with patient enrollment and data collection		
Sara C	Martin		RN BSN	McGovern Medical School at The University of Texas Health Science Center at Houston	Houston, TX	assisted with patient enrollment and data collection		
Carrie M	Mason		MA LPA	McGovern Medical School at The University of Texas Health Science Center at Houston	Houston, TX	assisted with patient enrollment and data collection		
Georgia E	McDavid		RN	McGovern Medical School at The University of Texas Health Science Center at Houston	Houston, TX	assisted with patient enrollment and data collection		
Shannon L	McKee		EdS	McGovern Medical School at The University of Texas Health Science Center at Houston	Houston, TX	assisted with patient enrollment and data collection		
Michelle	Poe		PhD RN	McGovern Medical School at The University of Texas Health Science Center at Houston	Houston, TX	assisted with patient enrollment and data collection		
Kimberly	Rennie		PhD	McGovern Medical School at The University of Texas Health Science Center at Houston	Houston, TX	assisted with patient enrollment and data collection		
Shawna L	Rodgers		RNC-NIC BSN	McGovern Medical School at The University of Texas Health Science Center at Houston	Houston, TX	assisted with patient enrollment and data collection		
Saba Khan	Siddiki		MD	McGovern Medical School at The University of Texas Health Science Center at Houston	Houston, TX	investigator		
Daniel	Sperry		RN	McGovern Medical School at The University of Texas Health Science Center at Houston	Houston, TX	assisted with patient enrollment and data collection		

*First name, last name, and suffix (if applicable) are required and will appear in PubMed.

***First Name and Middle Initial(s)**	***Last Name**	***Suffix (eg, Jr, III)**	Academic Degrees	Institution	Location (city, state/province, country)	Role or Contribution, eg, chair, principal investigator	Group (if more than 1 Group listed in the byline) and/or Subgroup (eg, Steering Committee)	
Emily K	Stephens		RNC-NIC BSN	McGovern Medical School at The University of Texas Health Science Center at Houston	Houston, TX	assisted with patient enrollment and data collection		
Patti L	Pierce Tate		RCP	McGovern Medical School at The University of Texas Health Science Center at Houston	Houston, TX	assisted with patient enrollment and data collection		
Sharon L	Wright		MT (ASCP)	University of Texas Health Science	Houston, TX	assisted with patient		
Pablo J	Sanchez		MD	Nationwide Children's Hospital	Columbus, OH	investigator		
Leif D	Nelin		MD	Nationwide Children's Hospital	Columbus, OH	investigator		
Sudarshan R	Jadcherla		MD	Nationwide Children's Hospital	Columbus, OH	investigator		
Jonathan L	Slaughter		MD MPH	Nationwide Children's Hospital	Columbus, OH	investigator		
Patricia W	Luzader		RN	Nationwide Children's Hospital	Columbus, OH	assisted with patient enrollment and data collection		
Stephanie	Burkhardt		BS MPH	Nationwide Children's Hospital	Columbus, OH	assisted with patient enrollment and data collection		
Helen	Carey		PT DHSc PCS	Nationwide Children's Hospital	Columbus, OH	assisted with patient enrollment and data collection		
Michelle	Chao		BS	Nationwide Children's Hospital	Columbus, OH	assisted with patient enrollment and data collection		
Erna	Clark		BA	Nationwide Children's Hospital	Columbus, OH	assisted with patient enrollment and data collection		
Erin	Fearns			Nationwide Children's Hospital	Columbus, OH	assisted with patient enrollment and data collection		
Christine A	Fortney		PhD RN	Nationwide Children's Hospital	Columbus, OH	assisted with patient enrollment and data collection		

Supplemental Online Content: Nonauthor Collaborators
*First name, last name, and suffix (if applicable) are required and will appear in PubMed.

***First Name and Middle Initial(s)**	***Last Name**	***Suffix (eg, Jr, III)**	Academic Degrees	Institution	Location (city, state/province, country)	Role or Contribution, eg, chair, principal investigator	Group (if more than 1 Group listed in the byline) and/or Subgroup (eg, Steering Committee)	
Aubrey	Fowler		BS	Nationwide Children's Hospital	Columbus, OH	assisted with patient enrollment and data collection		
Jennifer	Grothause		RN BSN	Nationwide Children's Hospital	Columbus, OH	assisted with patient enrollment and data collection		
Julie	Gutentag		RN BSN	Nationwide Children's Hospital	Columbus, OH	assisted with patient enrollment and data collection		
Cole	Hague		BA MS	Nationwide Children's Hospital	Columbus, OH	assisted with patient enrollment and data collection		
Jacqueline	McCool			Nationwide Children's Hospital	Columbus, OH	assisted with patient enrollment and data collection		
Mary Ann	Nelin		MD	Nationwide Children's Hospital	Columbus, OH	investigator		
Courtney	Park		RN BSN	Nationwide Children's Hospital	Columbus, OH	assisted with patient enrollment and data collection		
Lindsay	Pietruszewski		PTN DPT	Nationwide Children's Hospital	Columbus, OH	assisted with patient enrollment and data collection		
Jessica	Purnell		BS CCRC	Nationwide Children's Hospital	Columbus, OH	assisted with patient enrollment and data collection		
Julie	Shadd		BS RD LD	Nationwide Children's Hospital	Columbus, OH	assisted with patient enrollment and data collection		
Kristi	Small		BS	Nationwide Children's Hospital	Columbus, OH	assisted with patient enrollment and data collection		

Supplemental Online Content: Nonauthor Collaborators
*First name, last name, and suffix (if applicable) are required and will appear in PubMed.

*First Name and Middle Initial(s)	*Last Name	*Suffix (eg, Jr, III)	Academic Degrees	Institution	Location (city, state/province, country)	Role or Contribution, eg, chair, principal investigator	Group (if more than 1 Group listed in the byline) and/or Subgroup (eg, Steering Committee)	
Melanie	Stein		RRT BBA	Nationwide Children's Hospital	Columbus, OH	assisted with patient enrollment and data collection		
Margaret	Sullivan		BS	Nationwide Children's Hospital	Columbus, OH	assisted with patient enrollment and data collection		
Rox Ann	Sullivan		RN BSN	Nationwide Children's Hospital	Columbus, OH	assisted with patient enrollment and data collection		
Christopher J	Timan		MD	Nationwide Children's Hospital	Columbus, OH	investigator		
Keith O	Yeates		MD PhD	Nationwide Children's Hospital	Columbus, OH	investigator		
Lina	Yoseff-Salameh		MD	Nationwide Children's Hospital	Columbus, OH	investigator		
Sarah A	Keim		PhD	Nationwide Children's Hospital	Columbus, OH	assisted with patient enrollment and data collection		
Julia	Newton		MPH	Nationwide Children's Hospital	Columbus, OH	assisted with patient enrollment and data collection		
Katelyn	Levengood		PT DPT	Nationwide Children's Hospital	Columbus, OH	assisted with patient enrollment and data collection		
Nancy	Batterson		OT/L SCFES CLC	Nationwide Children's Hospital	Columbus, OH	assisted with patient enrollment and data collection		
Courtney	Rice		PsyD	Nationwide Children's Hospital	Columbus, OH	assisted with patient enrollment and data collection		
Mohannad	Moallem		MD	Nationwide Children's Hospital	Columbus, OH	investigator		
Nathalie L	Maitre		MD PhD	Nationwide Children's Hospital	Columbus, OH	investigator		
Heidi M	Harmon		MD MS	Nationwide Children's Hospital	Columbus, OH	investigator		

Supplemental Online Content: Nonauthor Collaborators
*First name, last name, and suffix (if applicable) are required and will appear in PubMed.

***First Name and Middle Initial(s)**	***Last Name**	***Suffix (eg, Jr, III)**	Academic Degrees	Institution	Location (city, state/province, country)	Role or Contribution, eg, chair, principal investigator	Group (if more than 1 Group listed in the byline) and/or Subgroup (eg, Steering Committee)	
Abhik	Das		PhD	RTI International	Research Triangle Park, NC	assisted with patient enrollment and data collection		
Dennis	Wallace		PhD	RTI International	Research Triangle Park, NC	assisted with patient enrollment and data collection		
Carla M	Banna		PhD	RTI International	Research Triangle Park, NC	assisted with patient enrollment and data collection		
Marie G	Gantz		PhD	RTI International	Research Triangle Park, NC	assisted with patient enrollment and data collection		
Jeanette	O'Donnell Auman		BS	RTI International	Research Triangle Park, NC	assisted with patient enrollment and data collection		
Margaret M	Crawford		BS CCRP	RTI International	Research Triangle Park, NC	assisted with patient enrollment and data collection		
Jenna	Gabrio		MPH	RTI International	Research Triangle Park, NC	assisted with patient enrollment and data collection		
David	Leblond		BS	RTI International	Research Triangle Park, NC	assisted with patient enrollment and data collection		
Jamie E	Newman		PhD MPH	RTI International	Research Triangle Park, NC			
Carolyn M	Petrie Huitema		MS CCRP	RTI International	Research Triangle Park, NC	assisted with patient enrollment and data collection		
Annie	vonLehmden		BS	RTI International	Research Triangle Park, NC	assisted with patient enrollment and data collection		
Kristin M	Zaterka-Baxter		RN BSN CCRP	RTI International	Research Triangle Park, NC	assisted with patient enrollment and data collection		
Lei	Li		PhD	RTI International	Research Triangle Park, NC	assisted with patient enrollment and data collection		

*First name, last name, and suffix (if applicable) are required and will appear in PubMed.

***First Name and Middle Initial(s)**	***Last Name**	***Suffix (eg, Jr, III)**	Academic Degrees	Institution	Location (city, state/province, country)	Role or Contribution, eg, chair, principal investigator	Group (if more than 1 Group listed in the byline) and/or Subgroup (eg, Steering Committee)	
Krisa P	Van Meurs		MD	Stanford University	Stanford, CA	investigator		
David K	Stevenson		MD	Stanford University	Stanford, CA	investigator		
Valerie Y	Chock		MD MS Epi	Stanford University	Stanford, CA	investigator		
M. Bethany	Ball		BSc CCRC	Stanford University	Stanford, CA	assisted with patient enrollment and data collection		
Barbara	Bentley		PsychD MSEd	Stanford University	Stanford, CA	assisted with patient enrollment and data collection		
Ritu	Chitkara		MD FAAP	Stanford University	Stanford, CA	investigator		
Alexis S	Davis		MD	Stanford University	Stanford, CA	investigator		
Maria Elena	DeAnda		PhD	Stanford University	Stanford, CA	assisted with patient enrollment and data collection		
Anne M	DeBattista		RN PNP-C PhD	Stanford University	Stanford, CA	assisted with patient enrollment and data collection		
Beth	Earhart		PhD	Stanford University	Stanford, CA	assisted with patient enrollment and data collection		
Lynne C	Huffman		MD	Stanford University	Stanford, CA	investigator		
Casey E	Krueger		PhD	Stanford University	Stanford, CA	assisted with patient enrollment and data collection		
Ryan E	Lucash		PhD	Stanford University	Stanford, CA	assisted with patient enrollment and data collection		
Melinda S	Proud		RCP	Stanford University	Stanford, CA	assisted with patient enrollment and data collection		
Elizabeth N	Hitchner Reichert		MA CCRC	Stanford University	Stanford, CA	assisted with patient enrollment and data collection		
Dharshi	Sivakumar		MD	Stanford University	Stanford, CA	investigator		
Heather	Taylor		PhD	Stanford University	Stanford, CA	assisted with patient enrollment and data collection		
Hali E	Weiss		MD	Stanford University	Stanford, CA	investigator		
Myriam	Peralta-Carcelen		MD MPH	University of Alabama at Birmingham	Birmingham, AL	investigator		
Monica V	Collins		RN BSN	University of Alabama at Birmingham	Birmingham, AL	assisted with patient enrollment and data collection		
Shirley S	Cosby		RN BSN	University of Alabama at Birmingham	Birmingham, AL	assisted with patient enrollment and data collection		
Fred J	Biasini		PhD	University of Alabama at Birmingham	Birmingham, AL	assisted with patient enrollment and data collection		
Kristy A	Domnanovich		PhD	University of Alabama at Birmingham	Birmingham, AL	assisted with patient enrollment and data collection		

*First name, last name, and suffix (if applicable) are required and will appear in PubMed.

*First Name and Middle Initial(s)	*Last Name	*Suffix (eg, Jr, III)	Academic Degrees	Institution	Location (city, state/province, country)	Role or Contribution, eg, chair, principal investigator	Group (if more than 1 Group listed in the byline) and/or Subgroup (eg, Steering Committee)	
Tara E	McNair		RN BSN	University of Alabama at Birmingham	Birmingham, AL	assisted with patient enrollment and data collection		
Vivien A	Phillips		RN BSN	University of Alabama at Birmingham	Birmingham, AL	assisted with patient enrollment and data collection		
Sally	Whitley		MA OTR-L FAOTA	University of Alabama at Birmingham	Birmingham, AL	assisted with patient enrollment and data collection		
Sheree	York Chapman		PT DPT PCS	University of Alabama at Birmingham	Birmingham, AL	assisted with patient enrollment and data collection		
Uday	Devaskar		MD	University of California - Los Angeles	Los Angeles, CA	investigator		
Teresa	Chanlaw		MPH	University of California - Los Angeles	Los Angeles, CA	assisted with patient enrollment and data collection		
Rachel	Geller		RN BSN	University of California - Los Angeles	Los Angeles, CA	assisted with patient enrollment and data collection		
Meena	Garg		MD	University of California - Los Angeles	Los Angeles, CA	investigator		
Isabell B	Purdy		PhD CPNP	University of California - Los Angeles	Los Angeles, CA	assisted with patient enrollment and data collection		
Edward F	Bell		MD	University of Iowa	Iowa City, IA	investigator		
Tarah T	Colaizy		MD MPH	University of Iowa	Iowa City, IA	investigator		
John A	Widness		MD	University of Iowa	Iowa City, IA	investigator		
Jane E	Brumbaugh		MD	University of Iowa	Iowa City, IA	investigator		
Heidi M	Harmon		MD	University of Iowa	Iowa City, IA	investigator		
Karen J	Johnson		RN BSN	University of Iowa	Iowa City, IA	assisted with patient enrollment and data collection		
Jacky R	Walker		RN	University of Iowa	Iowa City, IA	assisted with patient enrollment and data collection		
Claire A	Goeke		RN	University of Iowa	Iowa City, IA	assisted with patient enrollment and data collection		
Mendi L	Schmelzel		MSN RN	University of Iowa	Iowa City, IA	assisted with patient enrollment and data collection		
Diane L	Eastman		RN CPNP MA	University of Iowa	Iowa City, IA	assisted with patient enrollment and data collection		
Michelle L	Baack		MD	University of Iowa	Iowa City, IA	investigator		
Lauritz R	Meyer		MD	University of Iowa	Iowa City, IA	investigator		
Laurie A	Hogden		MD	University of Iowa	Iowa City, IA	investigator		
Megan M	Henning		RN	University of Iowa	Iowa City, IA	assisted with patient enrollment and data collection		

*First name, last name, and suffix (if applicable) are required and will appear in PubMed.

***First Name and Middle Initial(s)**	***Last Name**	***Suffix (eg, Jr, III)**	Academic Degrees	Institution	Location (city, state/province, country)	Role or Contribution, eg, chair, principal investigator	Group (if more than 1 Group listed in the byline) and/or Subgroup (eg, Steering Committee)	
Chelsey	Elenkiwich		RN BSN	University of Iowa	Iowa City, IA	assisted with patient enrollment and data collection		
Megan	Broadbent		RN BSN	University of Iowa	Iowa City, IA	assisted with patient enrollment and data collection		
Sarah	Van Muyden		RN BSN	University of Iowa	Iowa City, IA	assisted with patient enrollment and data collection		
Dan L	Ellsbury		MD	University of Iowa	Iowa City, IA	investigator		
Donia B	Campbell		RNC-NIC	University of Iowa	Iowa City, IA	assisted with patient enrollment and data collection		
Tracy L	Tud		RN	University of Iowa	Iowa City, IA	assisted with patient enrollment and data collection		
Janell	Fuller		MD	University of New Mexico Health Sciences Center	Albuquerque, NM	investigator		
Sundquist	Beauman		MSN RNC	University of New Mexico Health Sciences Center	Albuquerque, NM	assisted with patient enrollment and data collection		
Conra	Backstrom Lacy		RN	University of New Mexico Health Sciences Center	Albuquerque, NM	assisted with patient enrollment and data collection		
Mary	Ruffner Hanson		RN BSN	University of New Mexico Health Sciences Center	Albuquerque, NM	assisted with patient enrollment and data collection		
Carol	Hartenberger		BSN MPH	University of New Mexico Health Sciences Center	Albuquerque, NM	assisted with patient enrollment and data collection		
Elizabeth	Kuan		RN BSN	University of New Mexico Health Sciences Center	Albuquerque, NM	assisted with patient enrollment and data collection		
Sandra Jean R.	Lowe		PhD	University of New Mexico Health Sciences Center	Albuquerque, NM	assisted with patient enrollment and data collection		
Gregory M	Sokol		MD	Indiana University	Bloomington, IN	investigator		
Lu Ann	Papile		MD	Indiana University	Bloomington, IN	investigator		
Heidi	Harmon		MD, MS	Indiana University	Bloomington, IN	investigator		
Abbey C	Hines		PsyD HSPP	Indiana University	Bloomington, IN	assisted with patient enrollment and data collection		
Carolyn	Lytle		MD MPH	Indiana University	Bloomington, IN	investigator		
Dianne E	Herron		RN CCRC	Indiana University	Bloomington, IN	assisted with patient enrollment and data collection		
Susan	Gunn		NNP-BC CCRC	Indiana University	Bloomington, IN	assisted with patient enrollment and data collection		
Lucy	Smiley		CCRC	Indiana University	Bloomington, IN	assisted with patient enrollment and data collection		
Leslie	Dawn Wilson		BSN CCRC	Indiana University	Bloomington, IN	assisted with patient enrollment and data collection		
Sara B	DeMauro		MD MSCE	University of Pennsylvania	Philadelphia, PA	investigator		

Supplemental Online Content: Nonauthor Collaborators
*First name, last name, and suffix (if applicable) are required and will appear in PubMed.

*First Name and Middle Initial(s)	*Last Name	*Suffix (eg, Jr, III)	Academic Degrees	Institution	Location (city, state/province, country)	Role or Contribution, eg, chair, principal investigator	Group (if more than 1 Group listed in the byline) and/or Subgroup (eg, Steering Committee)	
Barbara	Schmidt		MD MSCE	University of Pennsylvania	Philadelphia, PA	investigator		
Eric C	Eichenwald		MD	University of Pennsylvania	Philadelphia, PA	investigator		
Haresh	Kirpalani		MD, MSc	University of Pennsylvania	Philadelphia, PA	investigator		
Sorava	Abbasi		MD	University of Pennsylvania	Philadelphia, PA	investigator		
Toni	Mancini		RN BSN CCRC	University of Pennsylvania	Philadelphia, PA	assisted with patient enrollment and data collection		
Aasma S	Chaudhary		BS RRT	University of Pennsylvania	Philadelphia, PA	assisted with patient enrollment and data collection		
Dara M	Cucinotta		RN	University of Pennsylvania	Philadelphia, PA	assisted with patient enrollment and data collection		
Judy C	Bernbaum		MD	University of Pennsylvania	Philadelphia, PA	investigator		
Andrea	Freeman Duncan		MD	University of Pennsylvania	Philadelphia, PA	investigator		
Kevin	Dysart		MD	University of Pennsylvania	Philadelphia, PA	investigator		
Marsha	Gerdes		PhD	University of Pennsylvania	Philadelphia, PA	assisted with patient enrollment and data collection		
Hallam	Hurt		MD	University of Pennsylvania	Philadelphia, PA	investigator		
Jonathan	Snyder		RN BSN	University of Pennsylvania	Philadelphia, PA	assisted with patient enrollment and data collection		
Kristina	Ziolkowski			University of Pennsylvania	Philadelphia, PA	assisted with patient enrollment and data collection		
Carl T	D'Angio		MD	University of Rochester	Rochester, NY	investigator		
Ronnie	Guillet		MD PhD	University of Rochester	Rochester, NY	investigator		
Gary J	Myers		MD	University of Rochester	Rochester, NY	investigator		
Kyle	Binion		BS	University of Rochester	Rochester, NY	assisted with patient enrollment and data collection		
Patricia R	Chess		MD	University of Rochester	Rochester, NY	investigator		
Caitlin	Fallone		MA	University of Rochester	Rochester, NY	assisted with patient enrollment and data collection		
Osman	Farooq		MD	University of Rochester	Rochester, NY	investigator		
Rosemary L Jensen				University of Rochester	Rochester, NY	assisted with patient enrollment and data collection		
Alison	Kent		BMBS FRACP MD	University of Rochester	Rochester, NY	assisted with patient enrollment and data collection		
Dee	Maffett		RN	University of Rochester	Rochester, NY	assisted with patient enrollment and data collection		
Joan	Merzbach		LMSW	University of Rochester	Rochester, NY	assisted with patient enrollment and data collection		
Constance	Orme			University of Rochester	Rochester, NY	assisted with patient enrollment and data collection		
Michael G	Sacilowski		BS	University of Rochester	Rochester, NY	assisted with patient enrollment and data collection		
Premini	Sabaratnam		MPH	University of Rochester	Rochester, NY	assisted with patient enrollment and data collection		

*First name, last name, and suffix (if applicable) are required and will appear in PubMed.

***First Name and Middle Initial(s)**	***Last Name**	***Suffix (eg, Jr, III)**	Academic Degrees	Institution	Location (city, state/province, country)	Role or Contribution, eg, chair, principal investigator	Group (if more than 1 Group listed in the byline) and/or Subgroup (eg, Steering Committee)	
Ann Marie	Scorsone		MS CCRC	University of Rochester	Rochester, NY	assisted with patient enrollment and data collection		
Holly I M	Wadkins			University of Rochester	Rochester, NY	assisted with patient enrollment and data collection		
Karen	Wynn		RN	University of Rochester	Rochester, NY	assisted with patient enrollment and data collection		
Kelley	Yost		PhD	University of Rochester	Rochester, NY	assisted with patient enrollment and data collection		
Anne Marie	Reynolds		MD	University of Rochester	Rochester, NY	investigator		
Satyan	Lakshminrusimha		MD	University of Rochester	Rochester, NY	investigator		
Praveen	Chandrasekharan		MD	University of Rochester	Rochester, NY	investigator		
Stephanie	Guilford		BS	University of Rochester	Rochester, NY	assisted with patient enrollment and data collection		
Michelle E	Hartley-McAndrews		MD	University of Rochester	Rochester, NY	investigator		
Ashley	Williams		MSEd	University of Rochester	Rochester, NY	assisted with patient enrollment and data collection		
William	Zorn		PhD	University of Rochester	Rochester, NY	assisted with patient enrollment and data collection		
Emily	Li		BA	University of Rochester	Rochester, NY	assisted with patient enrollment and data collection		
Jennifer	Donato		BS	University of Rochester	Rochester, NY	assisted with patient enrollment and data collection		
Kimberly G	McKee		BS	University of Rochester	Rochester, NY	assisted with patient enrollment and data collection		
Kelly R	Coleman		PsyD	University of Rochester	Rochester, NY	assisted with patient enrollment and data collection		
Stephen A	Bean		RPh	University of Rochester	Rochester, NY	assisted with patient enrollment and data collection		
Carol A	Coleman		RPh	University of Rochester	Rochester, NY	assisted with patient enrollment and data collection		
Cassandra A	Horihan		MS	University of Rochester	Rochester, NY	assisted with patient enrollment and data collection		
Myra H	Wyckoff		MD	University of Texas Southwestern Medical Center	Dallas, TX	investigator		
Luc P	Brion		MD	University of Texas Southwestern Medical Center	Dallas, TX	investigator		
Roy J	Heyne		MD	University of Texas Southwestern Medical Center	Dallas, TX	investigator		
Diana M	Vasil		MSN BSN RNC-NIC	University of Texas Southwestern Medical Center	Dallas, TX	assisted with patient enrollment and data collection		
Sally S	Adams		MS RN CPNP	University of Texas Southwestern Medical Center	Dallas, TX	assisted with patient enrollment and data collection		
Liiun	Chen		RN PhD	University of Texas Southwestern Medical Center	Dallas, TX	assisted with patient enrollment and data collection		

Supplemental Online Content: Nonauthor Collaborators
*First name, last name, and suffix (if applicable) are required and will appear in PubMed.

***First Name and Middle Initial(s)**	***Last Name**	***Suffix (eg, Jr, III)**	Academic Degrees	Institution	Location (city, state/province, country)	Role or Contribution, eg, chair, principal investigator	Group (if more than 1 Group listed in the byline) and/or Subgroup (eg, Steering Committee)	
Maria M	De Leon		RN BSN	University of Texas Southwestern Medical Center	Dallas, TX	assisted with patient enrollment and data collection		
Frances	Eubanks		RN BSN	University of Texas Southwestern Medical Center	Dallas, TX	assisted with patient enrollment and data collection		
Alicia	Guzman			University of Texas Southwestern Medical Center	Dallas, TX	assisted with patient enrollment and data collection		
Elizabeth	Heyne		PsyD PA-C	University of Texas Southwestern Medical Center	Dallas, TX	assisted with patient enrollment and data collection		
Lizette E	Lee		RN	University of Texas Southwestern Medical Center	Dallas, TX	assisted with patient enrollment and data collection		
Linda A	Madden		BSN RN CPNP	University of Texas Southwestern Medical Center	Dallas, TX	assisted with patient enrollment and data collection		
E Rebecca	McDougald		MSN APRN CPNP-PC/AC	University of Texas Southwestern Medical Center	Dallas, TX	assisted with patient enrollment and data collection		
Lara	Pavageau		MD	University of Texas Southwestern Medical Center	Dallas, TX	investigator		
Pollieanna	Sepulveda		RN	University of Texas Southwestern Medical Center	Dallas, TX	assisted with patient enrollment and data collection		
Cathy	Twell Boatman		MS CIMI	University of Texas Southwestern Medical Center	Dallas, TX	assisted with patient enrollment and data collection		
Kristine	Tolentino-Plata		MS	University of Texas Southwestern Medical Center	Dallas, TX	assisted with patient enrollment and data collection		
Azucena	Vera		AS	University of Texas Southwestern Medical Center	Dallas, TX	assisted with patient enrollment and data collection		
Jillian	Waterbury		DNP RN CPNP-PC	University of Texas Southwestern Medical Center	Dallas, TX	assisted with patient enrollment and data collection		
Bradley A	Yoder		MD	University of Utah Medical Center	Salt Lake City, UT	investigator		
Robin K	Ohls		MD	University of Utah Medical Center	Salt Lake City, UT	investigator		

*First name, last name, and suffix (if applicable) are required and will appear in PubMed.

***First Name and Middle Initial(s)**	***Last Name**	***Suffix (eg, Jr, III)**	Academic Degrees	Institution	Location (city, state/province, country)	Role or Contribution, eg, chair, principal investigator	Group (if more than 1 Group listed in the byline) and/or Subgroup (eg, Steering Committee)	
Mariana	Baserga		MD MSCI	University of Utah Medical Center	Salt Lake City, UT	investigator		
Sarah	Winter		MD	University of Utah Medical Center	Salt Lake City, UT	investigator		
Stephen D	Minton		MD	University of Utah Medical Center	Salt Lake City, UT	investigator		
Mark J	Sheffield		MD	University of Utah Medical Center	Salt Lake City, UT	investigator		
Carrie A	Rau		RN BSN CCRC	University of Utah Medical Center	Salt Lake City, UT	assisted with patient enrollment and data collection		
Shawna	Baker		NR	University of Utah Medical Center	Salt Lake City, UT	assisted with patient enrollment and data collection		
Jill	Burnett		RNC BSN	University of Utah Medical Center	Salt Lake City, UT	assisted with patient enrollment and data collection		
Susan	Christensen		RN	University of Utah Medical Center	Salt Lake City, UT	assisted with patient enrollment and data collection		
Laura	Cole Bledsoe		RN	University of Utah Medical Center	Salt Lake City, UT	assisted with patient enrollment and data collection		
Sean	Cunningham		PhD	University of Utah Medical Center	Salt Lake City, UT	assisted with patient enrollment and data collection		
Brandy	Davis		RN BSN	University of Utah Medical Center	Salt Lake City, UT	assisted with patient enrollment and data collection		
Jennifer O	Elmont		RN BSN	University of Utah Medical Center	Salt Lake City, UT	assisted with patient enrollment and data collection		
Becky	Hall		APRN	University of Utah Medical Center	Salt Lake City, UT	assisted with patient enrollment and data collection		
Manndi C	Loertscher		BS CCRP	University of Utah Medical Center	Salt Lake City, UT	assisted with patient enrollment and data collection		
Trisha	Marchant		RN	University of Utah Medical Center	Salt Lake City, UT	assisted with patient enrollment and data collection		
Earl	Maxon		RN CCRN	University of Utah Medical Center	Salt Lake City, UT	assisted with patient enrollment and data collection		

*First name, last name, and suffix (if applicable) are required and will appear in PubMed.

***First Name and Middle Initial(s)**	***Last Name**	***Suffix (eg, Jr, III)**	Academic Degrees	Institution	Location (city, state/province, country)	Role or Contribution, eg, chair, principal investigator	Group (if more than 1 Group listed in the byline) and/or Subgroup (eg, Steering Committee)	
Kandace M	McGrath			University of Utah Medical Center	Salt Lake City, UT	assisted with patient enrollment and data collection		
Hena G	Mickelsen		BA	University of Utah Medical Center	Salt Lake City, UT	assisted with patient enrollment and data collection		
Galina	Morshedzadeh		BSN APRN	University of Utah Medical Center	Salt Lake City, UT	assisted with patient enrollment and data collection		
D Melody	Parry		RN BSN	University of Utah Medical Center	Salt Lake City, UT	assisted with patient enrollment and data collection		
Brixen A	Reich		MSN RNC CCRC	University of Utah Medical Center	Salt Lake City, UT	assisted with patient enrollment and data collection		
Susan T	Schaefer		RN BSN RRT	University of Utah Medical Center	Salt Lake City, UT	assisted with patient enrollment and data collection		
Kelly	Stout		PhD	University of Utah Medical Center	Salt Lake City, UT	assisted with patient enrollment and data collection		
Ashley L	Stuart		PhD	University of Utah Medical Center	Salt Lake City, UT	assisted with patient enrollment and data collection		
Kimberlee	Weaver-Lewis		RN MS	University of Utah Medical Center	Salt Lake City, UT	assisted with patient enrollment and data collection		
Kathryn D	Woodbury		RN BSN	University of Utah Medical Center	Salt Lake City, UT	assisted with patient enrollment and data collection		
Seetha	Shankaran		MD	Wayne State University	Detroit, MI	investigator		
Sanjay	Chawla		MD	Wayne State University	Detroit, MI	investigator		
Girija	Natarajan		MD	Wayne State University	Detroit, MI	investigator		
Beena G	Sood		MD MS	Wayne State University	Detroit, MI	investigator		
Rebecca	Bara		RN BSN	Wayne State University	Detroit, MI	assisted with patient enrollment and data collection		
Prashant	Agarwal		MD	Wayne State University	Detroit, MI	investigator		
Monika	Bajaj		MD	Wayne State University	Detroit, MI	investigator		
Kirsten	Childs		RN BSN	Wayne State University	Detroit, MI	assisted with patient enrollment and data collection		
Melissa	February		MD	Wayne State University	Detroit, MI	investigator		
Laura	Goldston		MA	Wayne State University	Detroit, MI	assisted with patient enrollment and data collection		
Mary E	Johnson		RN BSN	Wayne State University	Detroit, MI	assisted with patient enrollment and data collection		
Bogdan	Panaitescu		MD PhD	Wayne State University	Detroit, MI	investigator		
Eunice	Hinz Woldt		RN MSN	Wayne State University	Detroit, MI	assisted with patient enrollment and data collection		

Supplemental Online Content: Nonauthor Collaborators
*First name, last name, and suffix (if applicable) are required and will appear in PubMed.

***First Name and Middle Initial(s)**	***Last Name**	***Suffix (eg, Jr, III)**	Academic Degrees	Institution	Location (city, state/province, country)	Role or Contribution, eg, chair, principal investigator	Group (if more than 1 Group listed in the byline) and/or Subgroup (eg, Steering Committee)	
John	Barks		MD	Wayne State University	Detroit, MI	investigator		
Martha	Carlson		MD	Wayne State University	Detroit, MI	investigator		
Mary K	Christensen		BA RRT	Wayne State University	Detroit, MI	investigator		
Diane F	White		RRT CCRP	Wayne State University	Detroit, MI	investigator		
Stephanie A	Wiggins		MS	Wayne State University	Detroit, MI	investigator		
