# Supplementary material for: Heterogeneity of Treatment Effects of Hydrocortisone by Risk of Bronchopulmonary Dysplasia or Death Among Extremely Preterm Infants in the National Institute of Child Health and Human Development Neonatal Research Network Trial: A Secondary Analysis of a Randomized Clinical Trial
Source: JAMA Netw Open. 2023 May 31;6(5):e2315315. doi: 10.1001/jamanetworkopen.2023.15315 (PMC10233424; doi:10.1001/jamanetworkopen.2023.15315)
Supplement: Supplement 4. — Data Sharing Statement [file jamanetwopen-e2315315-s004.pdf]

Data Sharing Statement

Gentle. Heterogeneity of Treatment Effects of Hydrocortisone by Risk of Bronchopulmonary Dysplasia or Death Among Extremely Preterm Infants in the National Institute of Child Health and Human Development Neonatal Research Network Trial. *JAMA Netw Open*. Published May 31, 2023. doi:10.1001/jamanetworkopen.2023.15315

Data
Data available: No
